# Supplementary material for: Structure function and engineering of multifunctional non-heme iron dependent oxygenases in fungal meroterpenoid biosynthesis
Source: Nat Commun. 2018 Jan 9;9:104. doi: 10.1038/s41467-017-02371-w (PMC5760668; doi:10.1038/s41467-017-02371-w)
Supplement: Supplementary file 1 — Supplementary Information [file 41467_2017_2371_MOESM1_ESM.pdf]

a AusE, PrhA-V150L/A232S/(M241V)

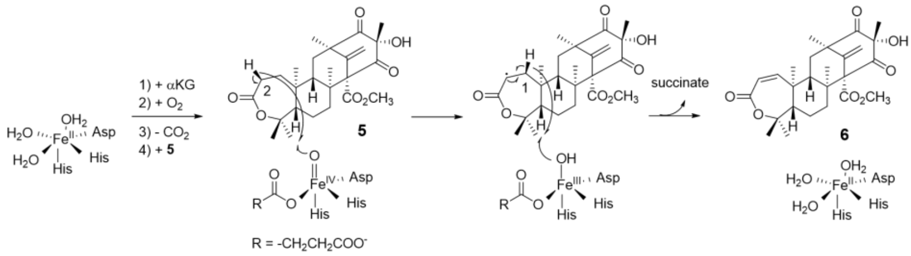

b AusE, PrhA-V150L/A232S/(M241V)

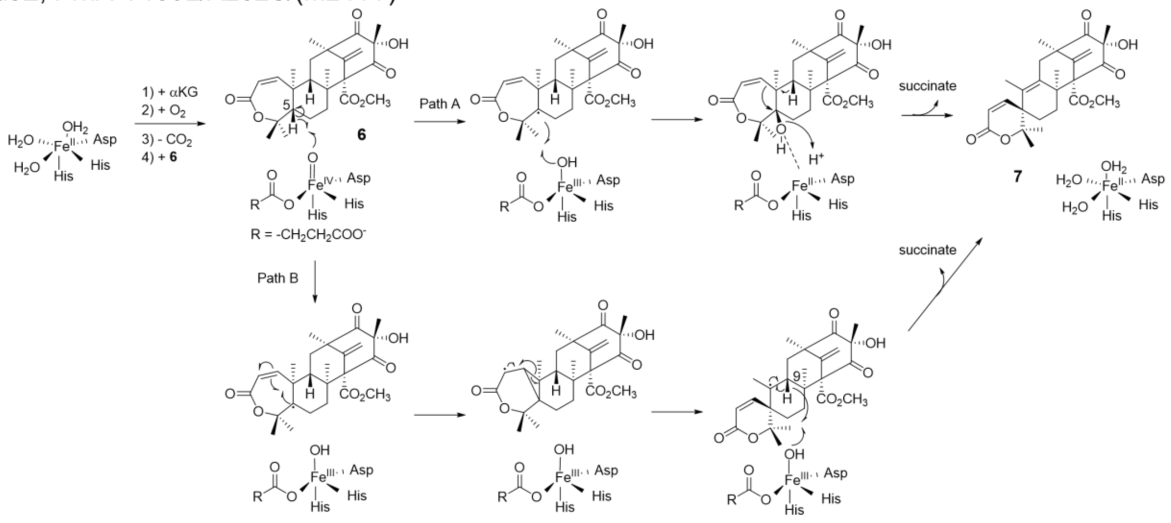

c PrhA, AusE-L150V/S232A

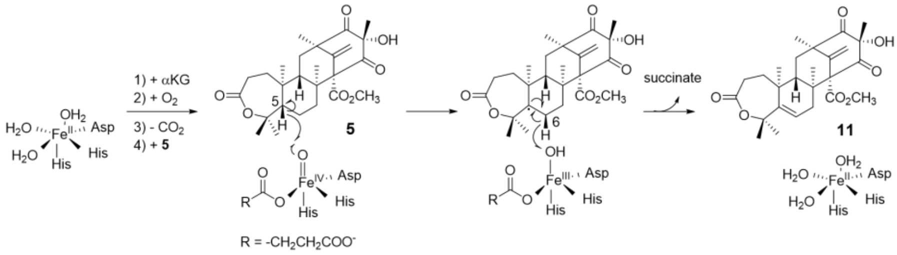

d AusE, PrhA-V150L/A232S/M241V

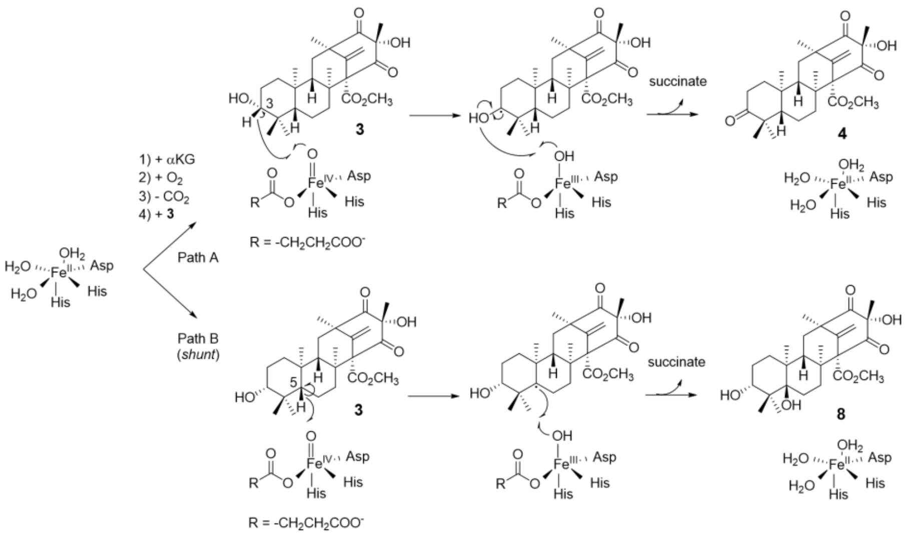

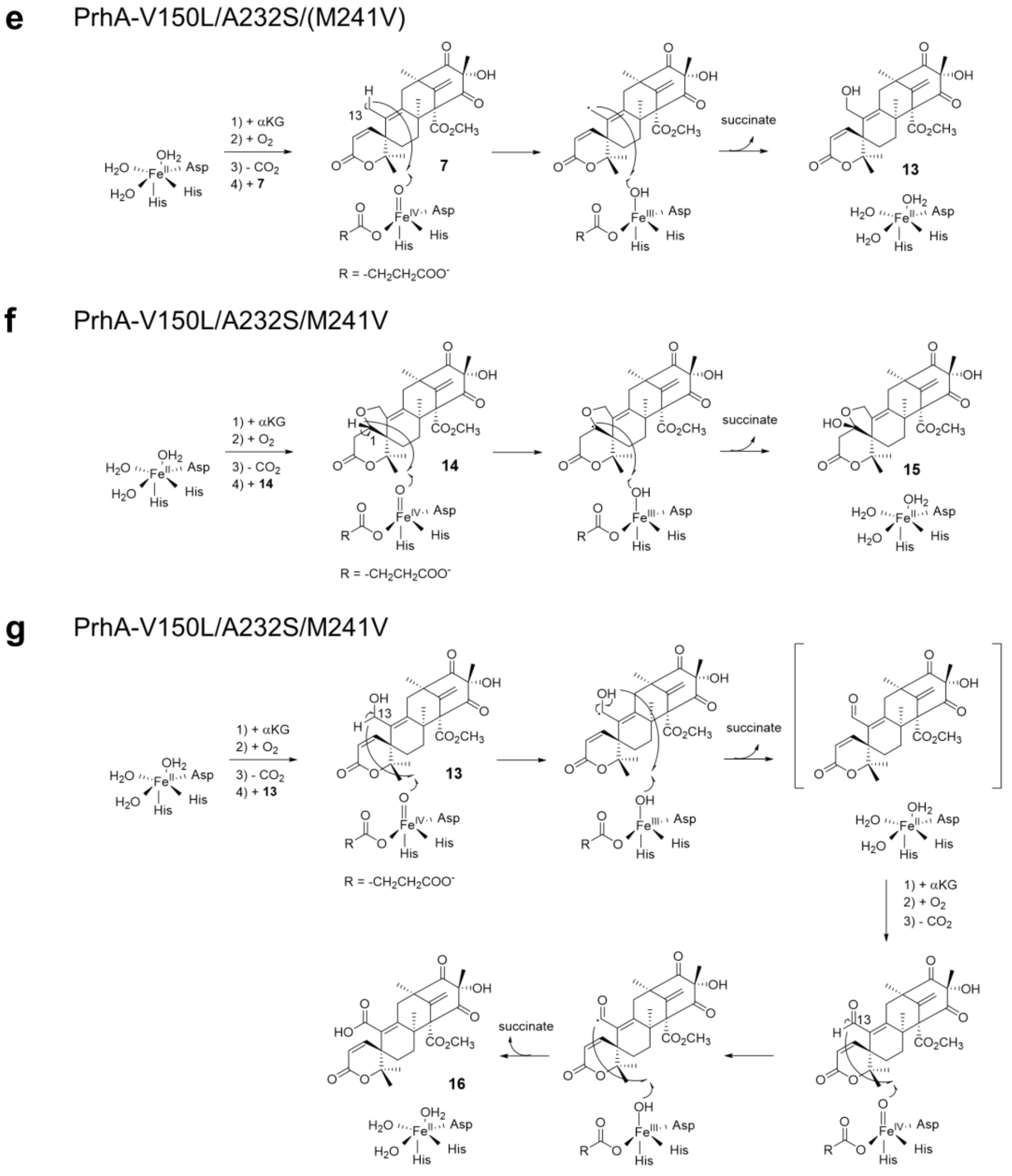

Supplementary Figure 1. Proposed mechanism for AusE, PrhA, and these mutants. (**a**) **5** is desaturated to form **6** through hydrogen atom abstraction at C-2 followed by the second hydrogen atom abstraction at C-1. (**b**) Conversion of **6** into **7** initiates with hydrogen atom abstraction at C-5. In path A, radical rebound from the top face results in the C-5 hydroxylation followed by rearrangement involving dehydration. This mechanism requires a base for the deprotonation of H-9. In path B, radical rearrangement results in cyclopropylcarbinyl radical formation, followed by ring opening to form the spirocyclic ring. The second hydrogen abstraction at C-9 forms the B-ring double bond. (**c**) Proposed mechanism for PrhA and AusE-L150V/S232A converting **5**

into **11** through hydrogen atom abstraction at C-5 and C-6. (**d**) AusE and PrhA-V150L/A232S/M241V convert **3** into **4** or **8** through initial hydrogen atom abstraction at C-3 or C-5, respectively. (**e**) PrhA-V150L/A232S/(M241V) converts **7** into **13** through initial hydrogen atom abstraction at C-13. (**f**) PrhA-V150L/A232S/M241V converts **14** into **15** through initial hydrogen atom abstraction at C-1. (**g**) PrhA-V150L/A232S/M241V converts **13** into **16** through initial hydrogen atom abstraction at C-13.

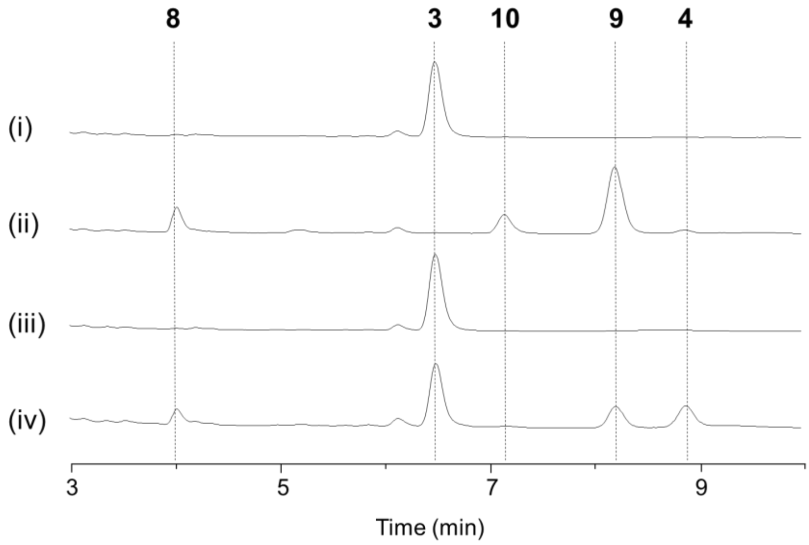

Supplementary Figure 2. Metal dependency of EDTA-treated AusE activity. HPLC profiles of the EDTA-treated AusE assays with **3**, αKG, and ascorbic acid: (**i**) without metal; (**ii**) with 10 mM $FeSO_4$; (**iii**) with 10 mM $MnCl_2$. (**iv**) HPLC profiles of the no-EDTA-treated assay with **3**, αKG, and ascorbic acid without metal. Chromatograms were monitored at 223 nm.

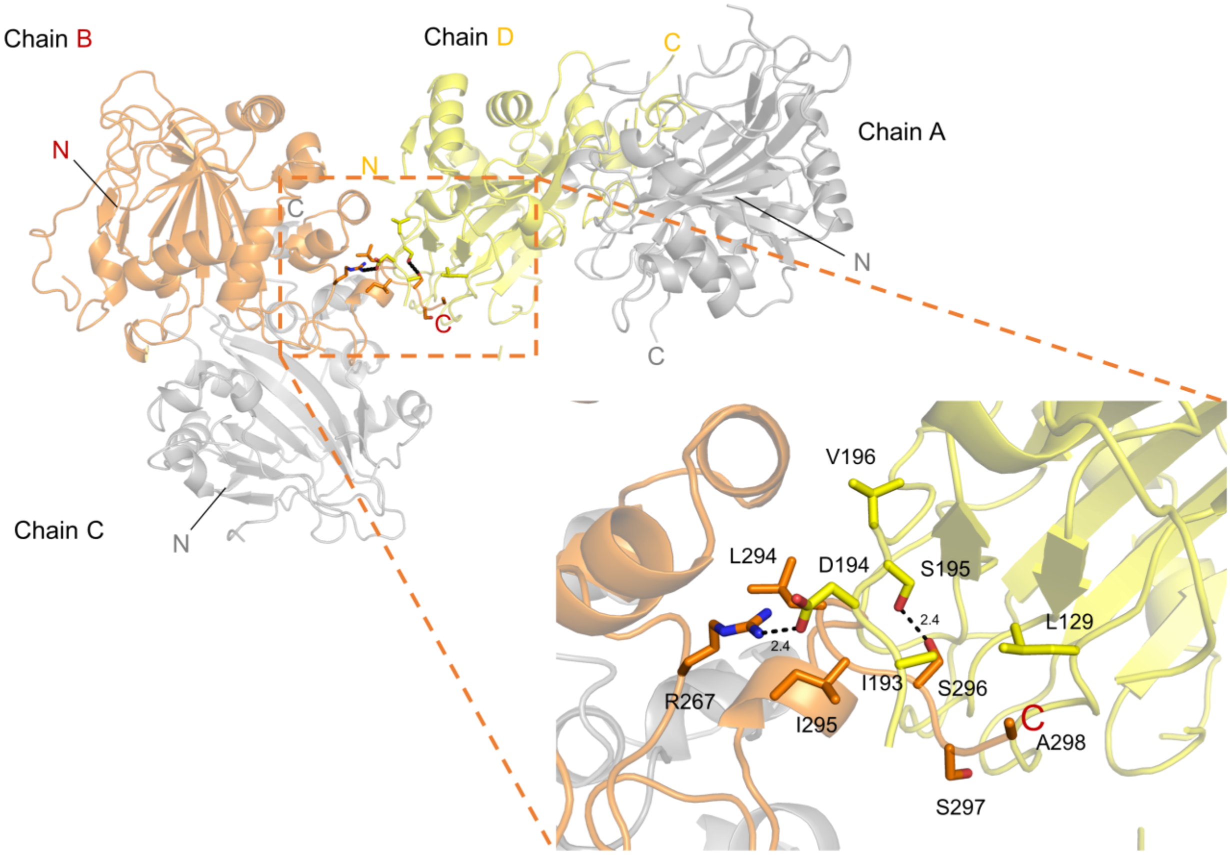

Supplementary Figure 3. Residues involved in inter-dimer interactions in AusE-Mn/αKG crystal. Amino acid residues located on the surface between chain B and chain D are shown as stick models. Hydrogen bonds were indicated as black dotted line. The positions of the N and C termini are indicated.

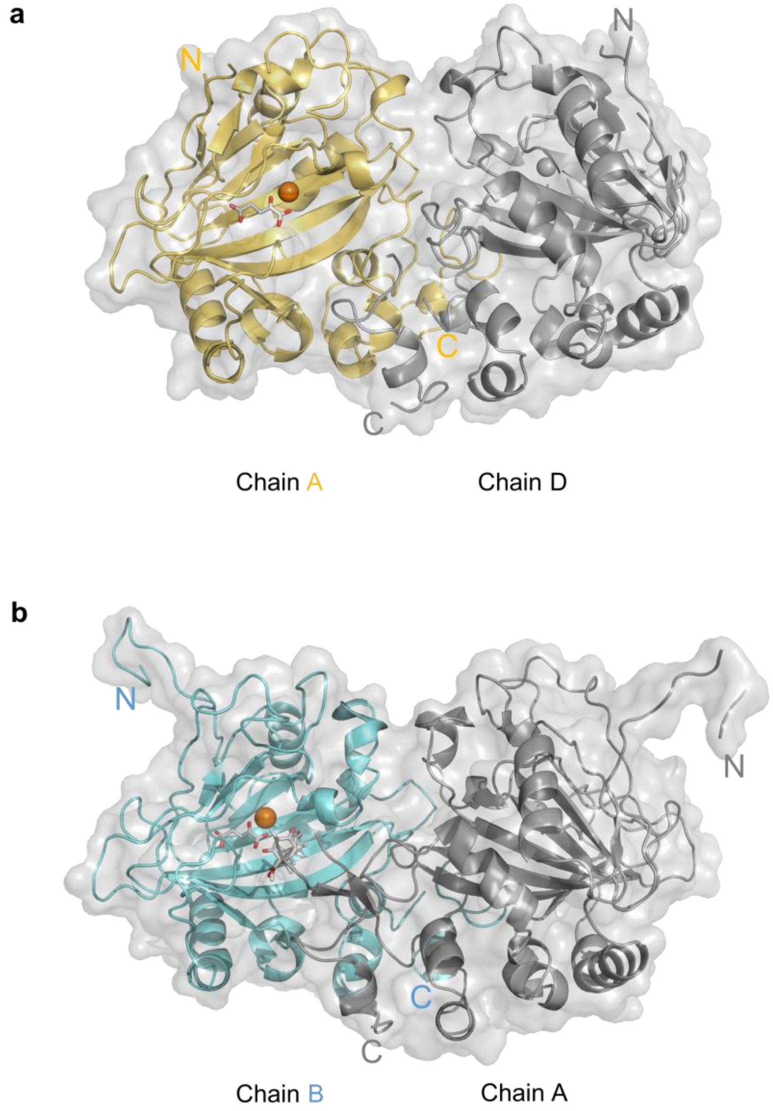

Supplementary Figure 4. Comparison of the overall structures and the active sites of AusE and PrhA. **(a-b)** The overall structures of **(a)** AusE and (**b**) PrhA with the positions of the N and C termini indicated. One of the monomeric subunits is colored in yellow (**a**), cyan (**b**), and the other is colored in gray. The co-substrate α-ketogluarate (αKG, white) and the substrate **5** (white) are shown as stick models.

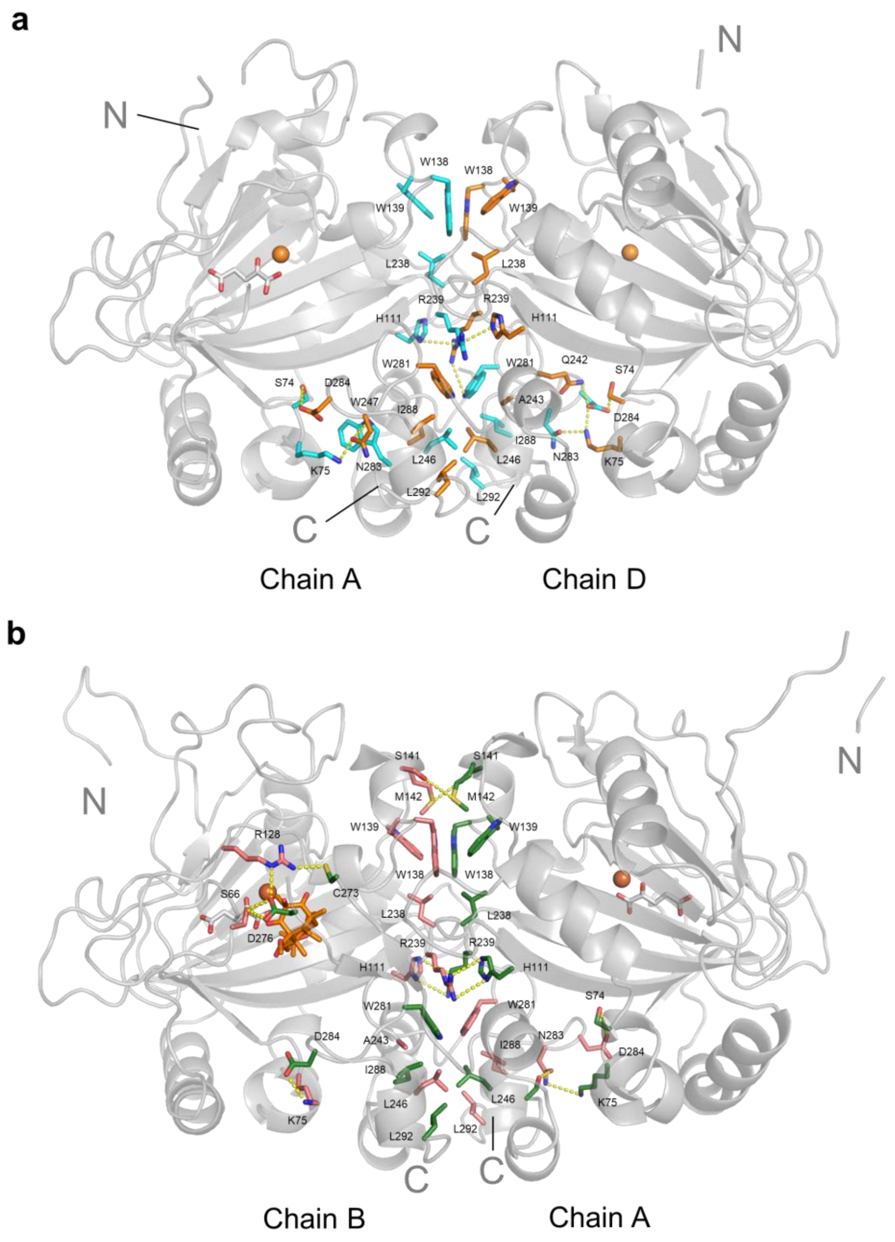

Supplementary Figure 5. The dimer interface of AusE and PrhA. (a) The dimer interface of AusE and **(b)** PrhA with residues in contact shown in sticks. The hydrogen bonds are represented as yellow dotted line. The positions of the N and C termini are indicated.

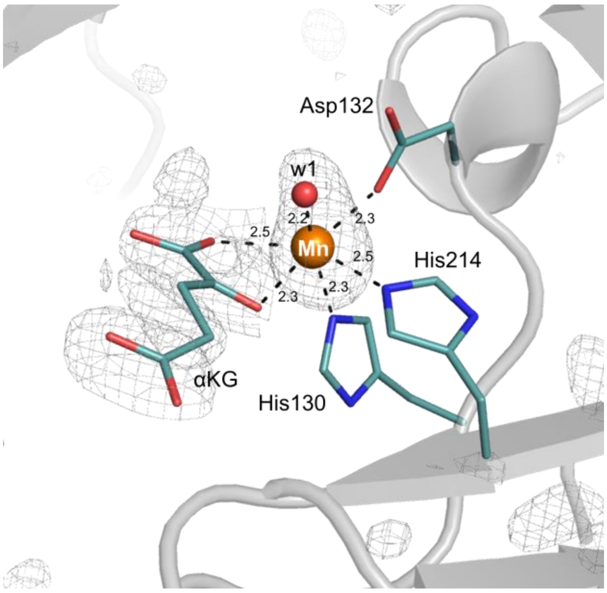

Supplementary Figure 6. Close-up views of the active site of AusE in complex with αKG. Structure shows the octahedral coordination of Mn^{2+} by the $HxDx_nH$ motif, the C-2 oxo group and C-1 carboxylate of αKG, and a water molecule (W). The *m*Fo-*D*Fc omit electron density map of Mn, w1, and αKG are represented as a black mesh, contoured at +3.5 σ. Interatomic distances are average values of Chain A and Chain C.

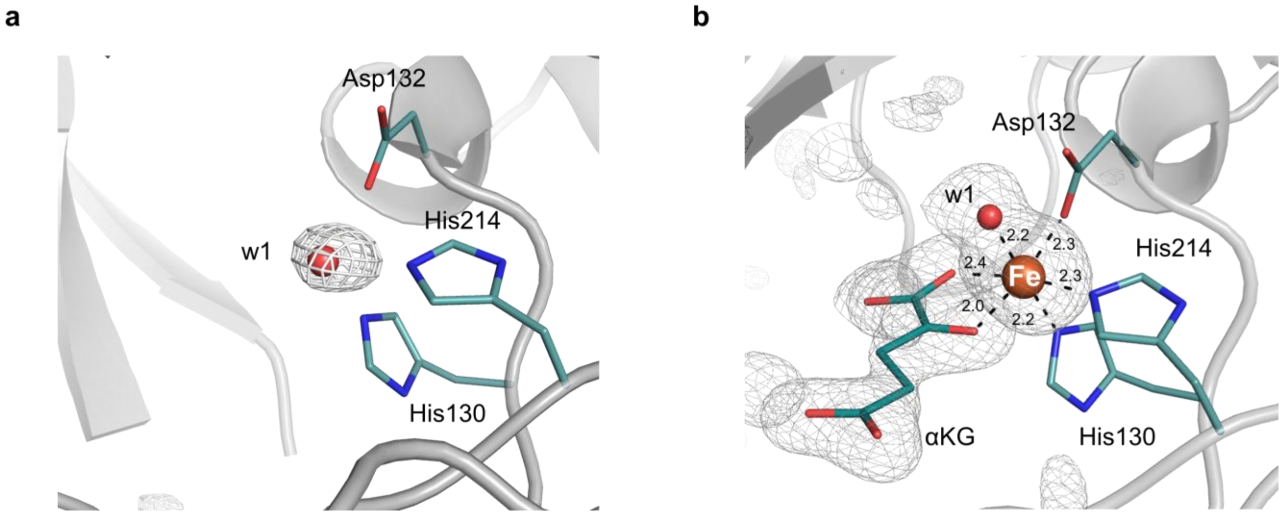

Supplementary Figure 7. Close-up views of the active site of apo PrhA and its complex structure with αKG. (a) The active site of the apo-state PrhA. A water molecule (W) was located in the center of $HxDx_nH$ motif. The *m*Fo-*D*Fc omit electron density map of w1 are represented as a black mesh, contoured at +6.0 σ. **(b)** The active site of PrhA-Fe(II)/αKG. The *m*Fo-*D*Fc omit electron density map of Fe, w1, and αKG are represented as a black mesh, contoured at +4.0 σ.

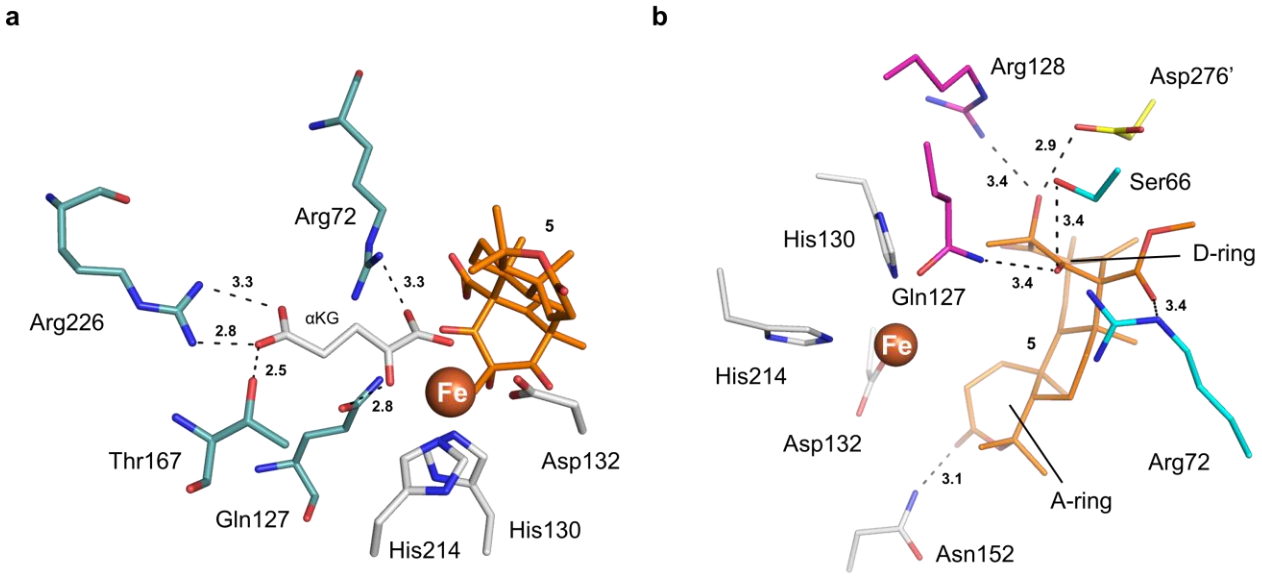

Supplementary Figure 8. Interaction of the active site residues with α-ketoglutarate (a) or preaustinoid A1 (5) (b) in PrhA-Fe/αKG/5 structure.

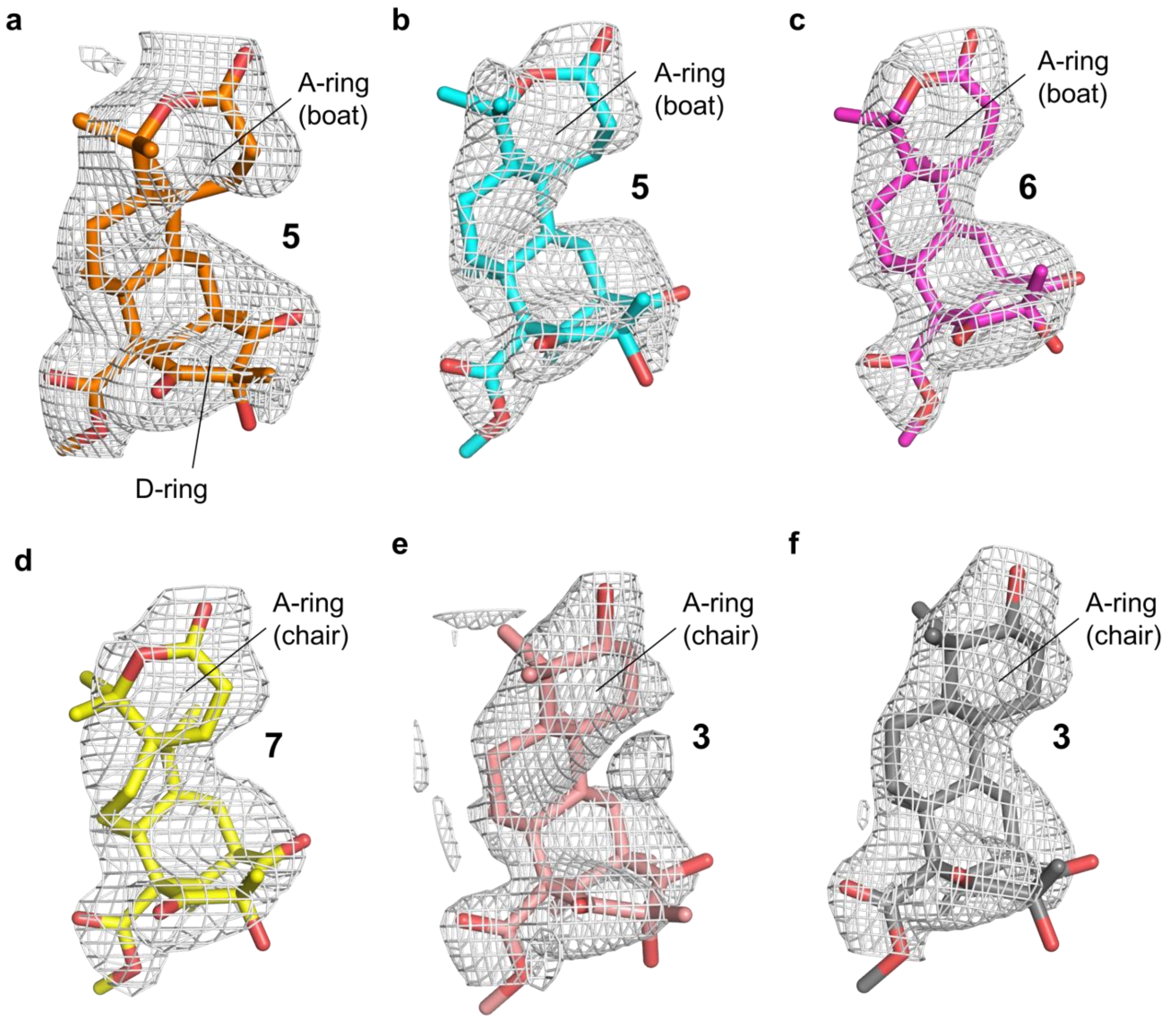

Supplementary Figure 9. Omit map of ligands in chain B. Close-up views of the ligand of PrhA in complex with (**a**) **5**, PrhA-V150L/A232S in complex with (**b**) **5**, (**c**) **6**, (**d**) **7**, and (**e**) **3**, PrhA-V150L/A232S/M241V in complex with (**f**) **3**. The *m*Fo-*D*Fc omit electron density maps of ligands are represented as a gray mesh, contoured at (**a**) +2.0 σ, (**b**) +2.0 σ, (**c**) +3.0 σ, (**d**) +3.0 σ, (**e**) +2.5 σ, and (**f**) +3.0 σ respectively.

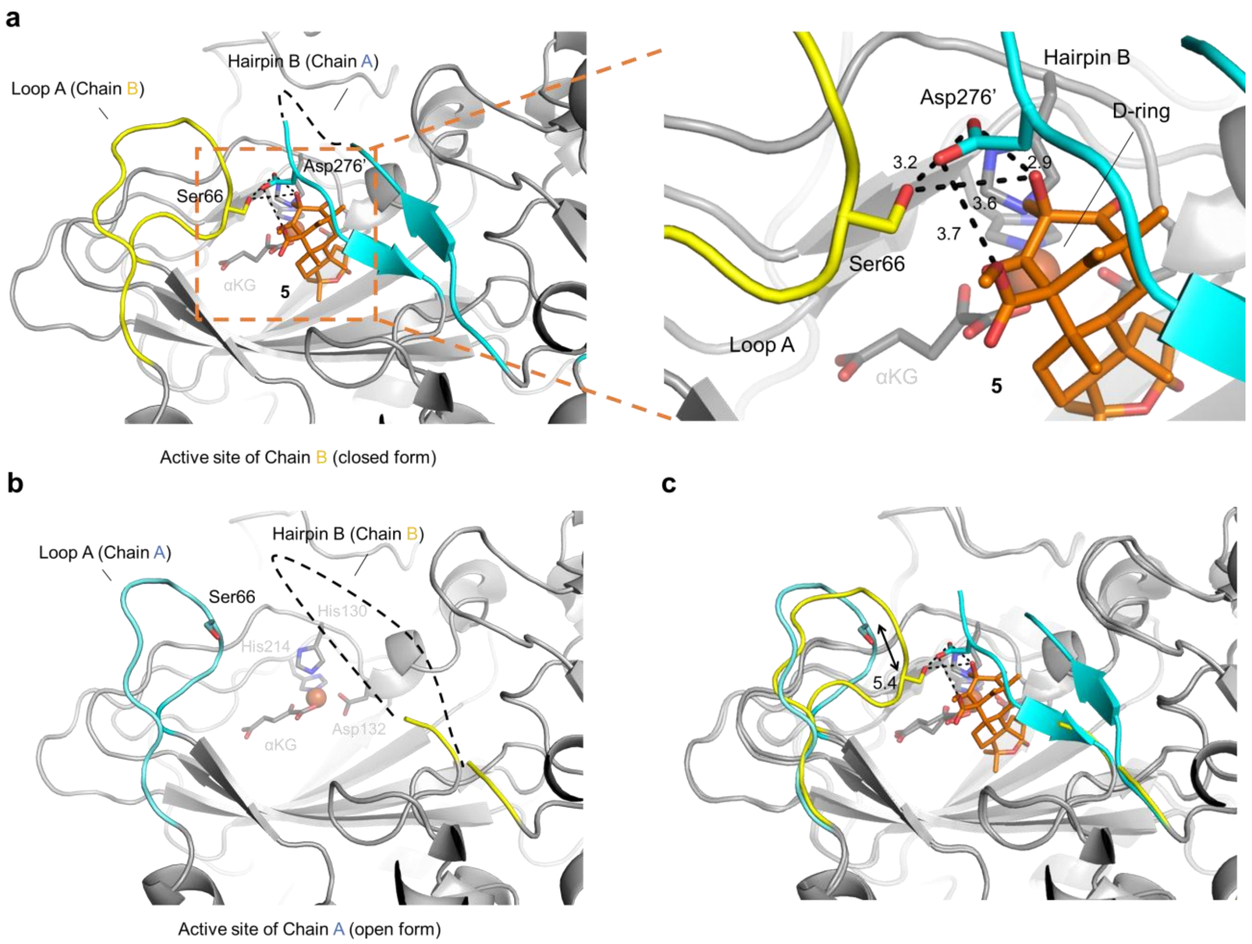

Supplementary Figure 10. C-terminal hairpin of chain A and the flexible loop form a lid in the active site of PrhA. (a) Active site of chain B contains ligand **5** (orange stick), which interacts with Loop A (yellow) of chain B and Hairpin B (cyan) of chain A (closed form). The side chains of Ser66 and Asp276' form hydrogen bond network with **5**. **(b)** In the case of chain A, the Loop A has moved away from the active site and the C-terminal hairpin of chain B is not observed in the absence of ligand **5** (open form). **(c)** Superimposition of the active sites of chain B **(a**, closed form**)** and chain A **(b**, open form**)**. Loop A has is 5.4 Å closer to the ligand **5** in chain B compared to chain A.

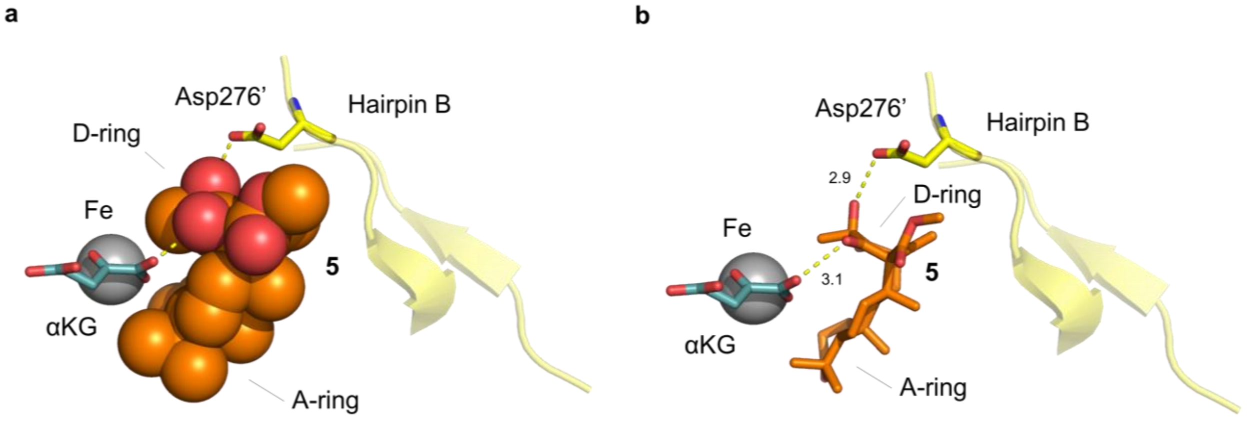

Supplementary Figure 11. The D-ring of 5 directly interacts with αKG and Hairpin B in PrhA-Fe/αKG/5 structure. (a) The substrate **5** shown as sphere model or **(b)** stick model. The substrate **5** is sandwiched between the αKG and Hairpin B with Asp276'. This interaction is likely to be important for substrate binding and release as αKG is consumed in each catalytic cycle.

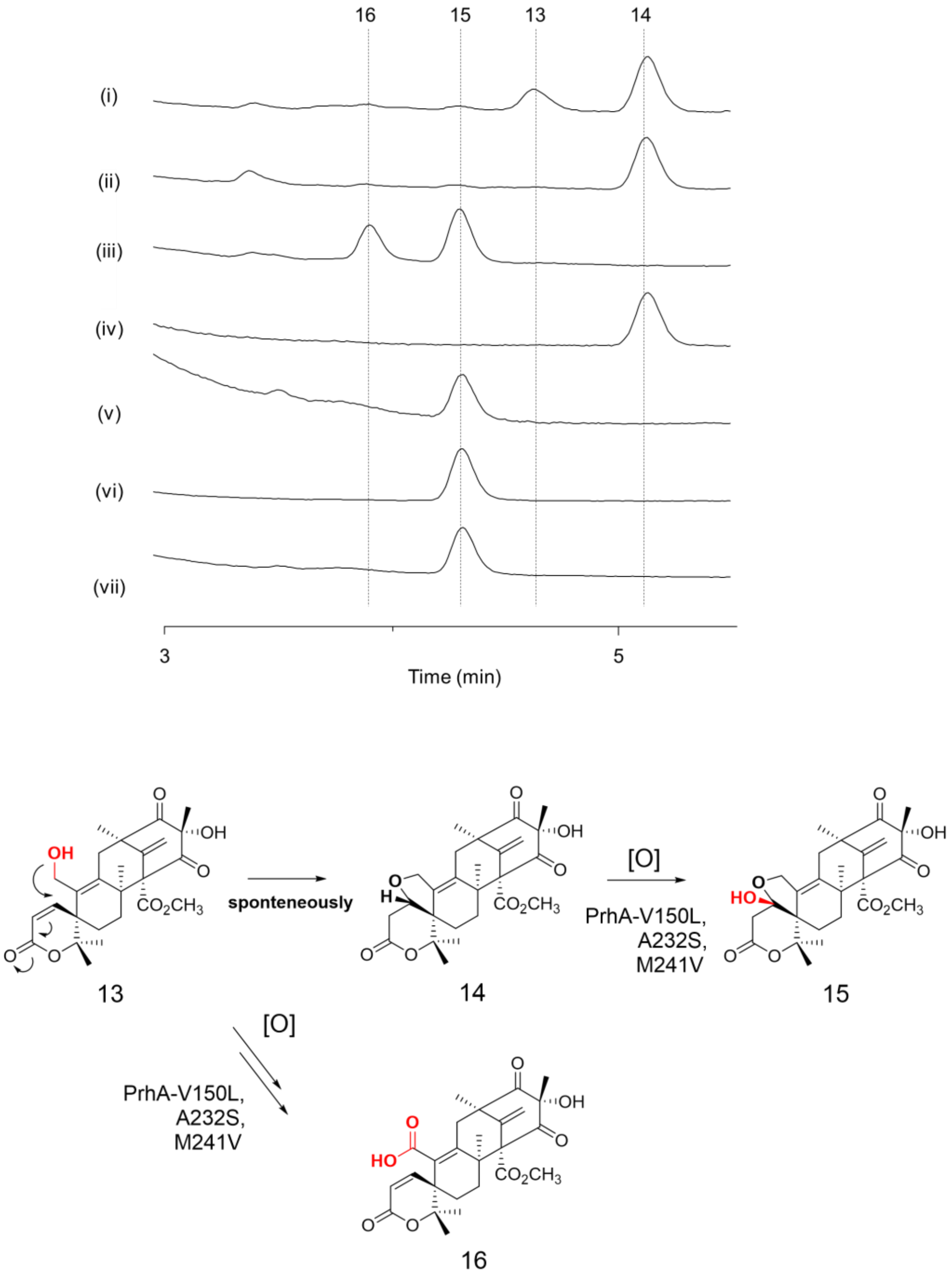

Supplementary Figure 12. *In vitro* enzyme reaction of PrhA-V150L/A232S/M241V. HPLC chromatograms of the assays performed with (**iii**) mixture of **13** and **14**, (**v**) **14**, and (**vii**) **15**. (**ii**) Mixture of **13** and **14**, (**iv**) **14**, or (**vi**) **15** incubated in pH7.5 PIPES buffer for 12 hr in the absence of PrhA-V150L/A232S/M241V were used as negative controls. (**i**) Isolated **13** spontaneously converts into **14** in the reaction buffer in the absence of enzyme. Chromatograms were monitored at 223 nm.

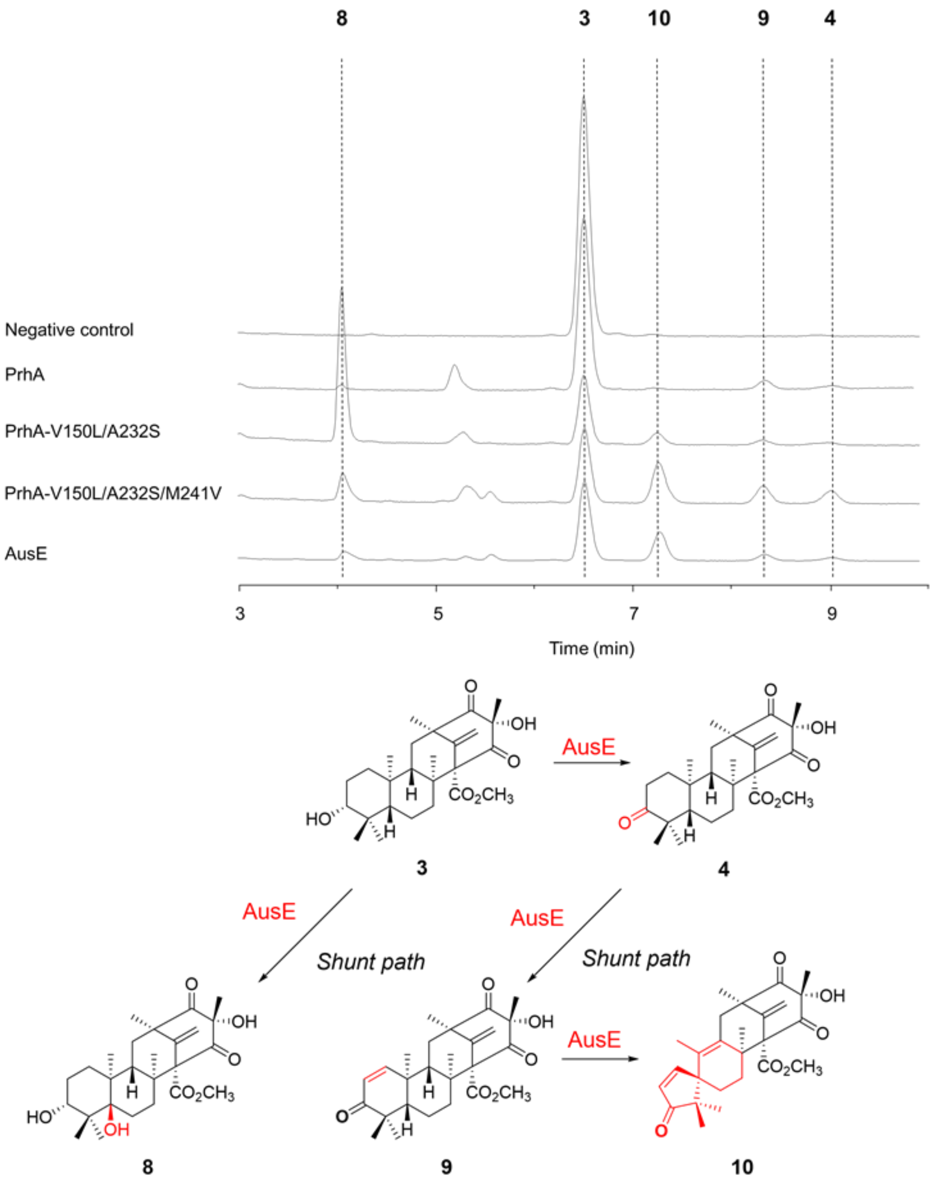

Supplementary Figure 13. ***In vitro*** **enzyme reaction of AusE, PrhA, and PrhA mutants with berkeleyone A (3). (a)** HPLC chromatograms of the productions of reactions of PrhA, PrhA-V150L/A232S, PrhA-V150L/A232S/M241V, and AusE. All of the reactions were performed for 1 hr. Chromatograms were monitored at 223 nm. AusE and PrhA mutants catalyze C-5 hydroxylation of **3** to form **8**, as well as desaturation and spirocycle formation to form **9** and **10** from **4**. This shunt activity is lacking in the wild type PrhA.

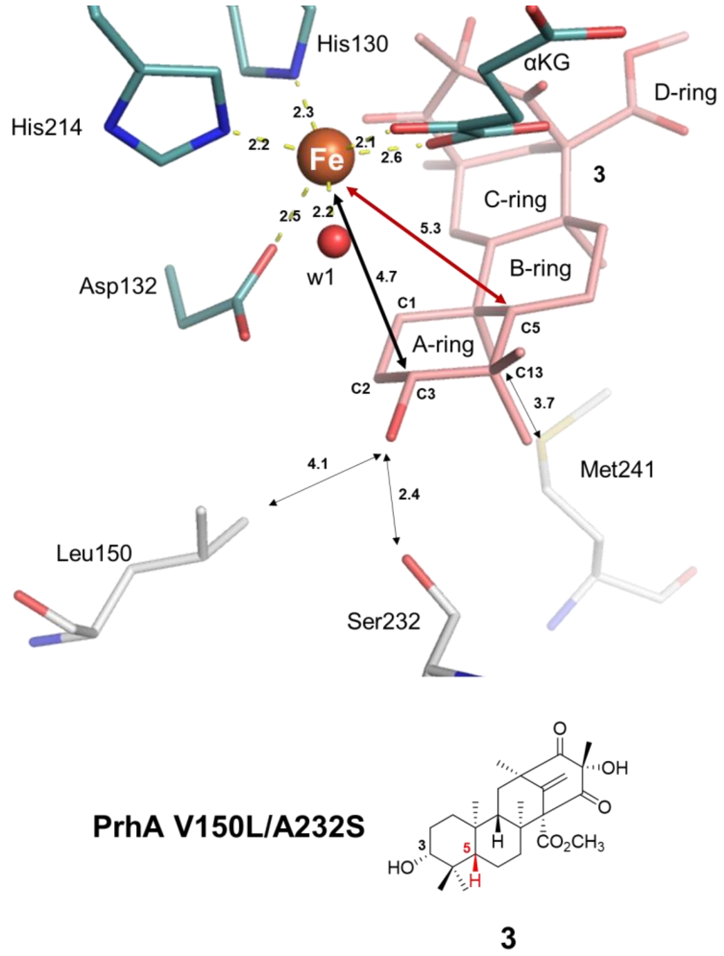

Supplementary Figure 14. Close-up views of the active site of PrhA(V150L/A232S)-Fe/αKG/**3.** Substrate **3** is colored as pink.

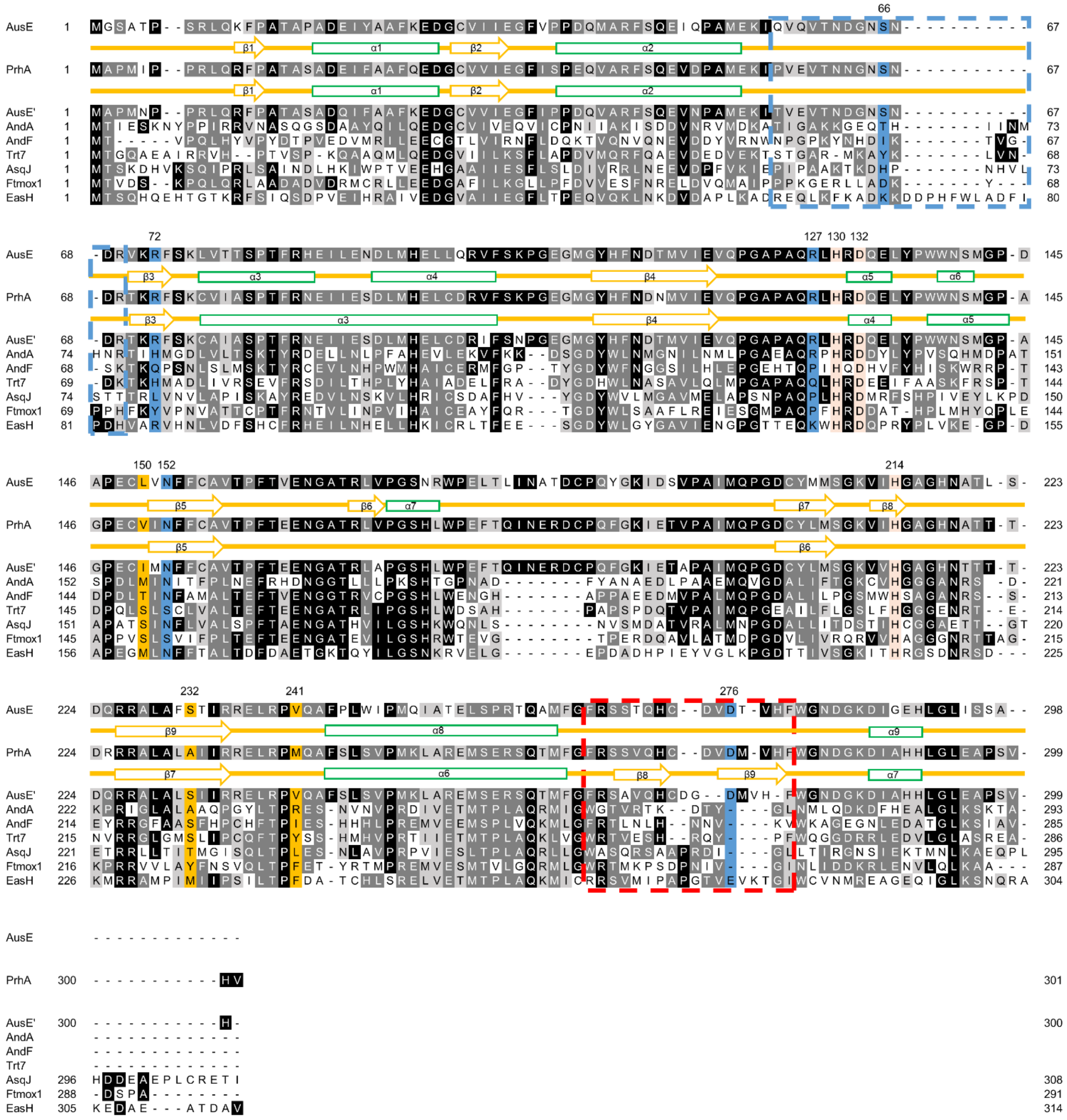

Supplementary Figure 15. Multiple sequence alignment of AusE, PrhA and their homologues. Primary sequence alignment of AusE and PrhA, AusE', AndA, AndF, AsqJ, and FtmOx1 with the highest sequence similarity found by Blast search. The secondary structures of AusE and PrhA are delineated as follows: α-helices (cylinders), β-strands (arrows). The HxDxnH motif highlighted in pink is strictly conserved for

Fe(II) binding. The three active site residues important for controlling the αKG oxygenase function (150, 232, and 241) are highlighted in yellow. Residues important for binding of the substrate highlighted in blue differ significantly among these homologues. Loop and hairpin regions constituting the substrate binding pocket are framed in blue and red dashed boxes, respectively. Accession numbers: AusE from *Aspergillus nidulans* FGSC A4 (Q5AR34.1), PrhA from *Penicillium brasilianum* (BAV69302.1), AusE' from *Penicillium brasilianum* MG11 (CEJ61311.1), AndA from *A. stellatus* (BAP81855.1), AndF from *A. stellatus* (BAP81860.1), Trt7 from *A. terreus* NIH2624 (Q0C8A0.1), AsqJ from *A. nidulans* FGSC A4 (Q5AR53.1), FtmOx1 from *A. fumigatus* Af293 (Q4WAW9.1), and EasH from *Claviceps purpurea* 20.1 (G8GV69.1).

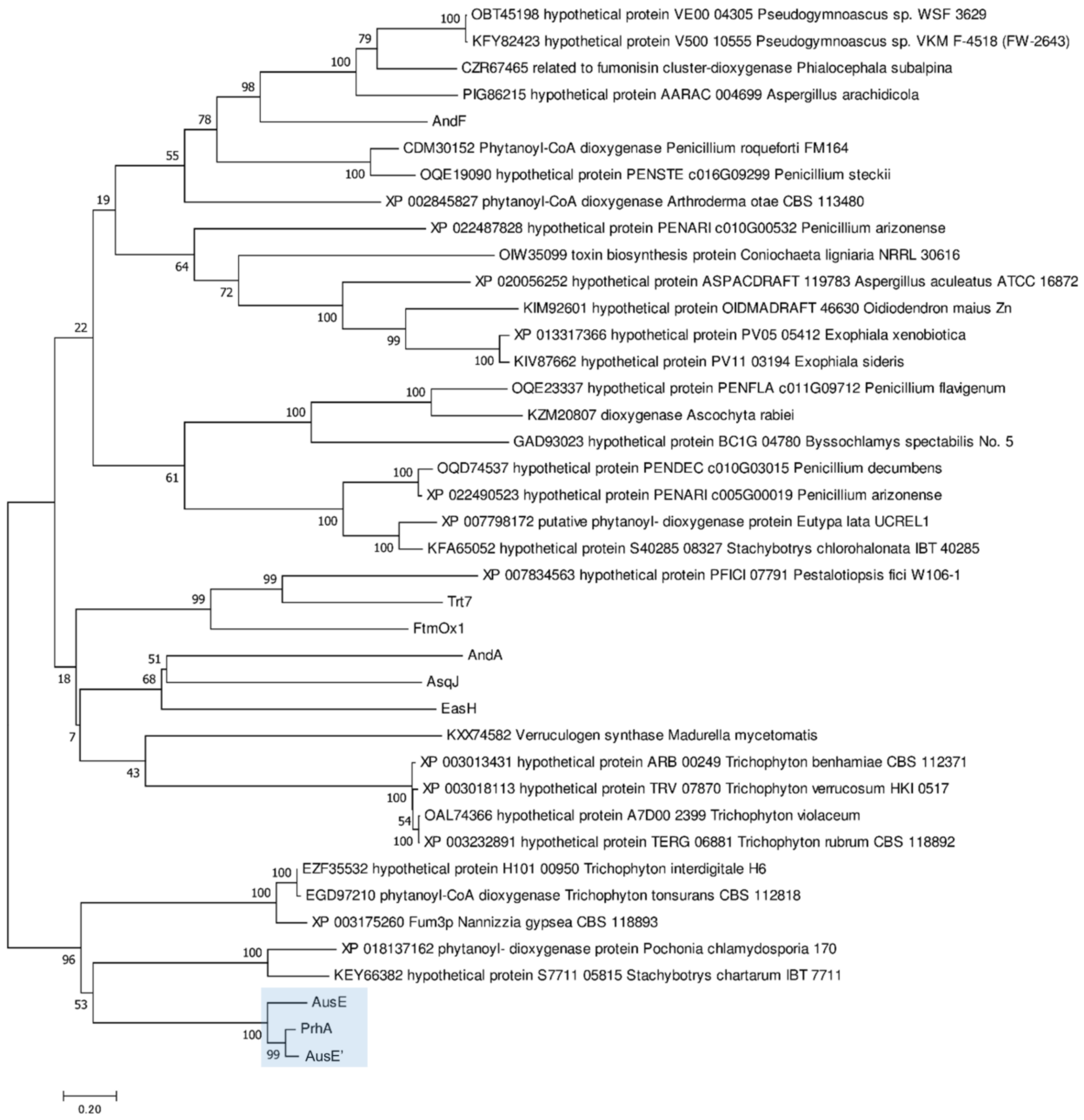

Supplementary Figure 16. Phylogenetic tree of AusE, PrhA and their homologues fungal non-heme iron oxygenases. Neighbor-joining method (MEGA 7) was used to generate this phylogenetic tree with a bootstrap test of 1,000 replicates. Bootstrap values given in percentages are shown at the nodes. AusE, AusE', and PrhA were surrounded by blue squares. These three enzymes are closely related. Accession numbers of AusE, PrhA, AusE', AndA, AndF, Trt7, AsqJ, FtmOx1, and EasH are the same as those used in Supplementary Figure 15.

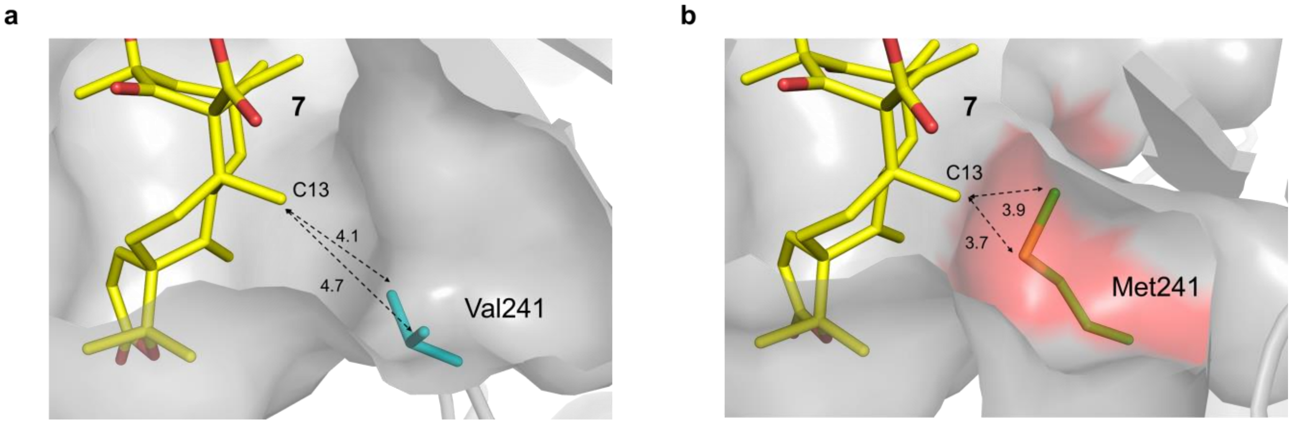

Supplementary Figure 17. M241V mutation in PrhA opens space for C-13 oxidation. (**a**) The **7** was manually docked into the active site of the PrhA-V150L/A232S/M241V triple mutant using the coordinates from the complex structure of **7** and the PrhA-V150L/A232S double mutant. Compared to (**b**) the complex structure of **7** and the PrhA-V150L/A232S double mutant, M241V mutation provides additional space around C-13 to allow further oxidation at this position. The surface of Met241 is highlighted in red with **7** and the relevant residues shown as stick models. The distance between the indicated atoms are provided in Å.

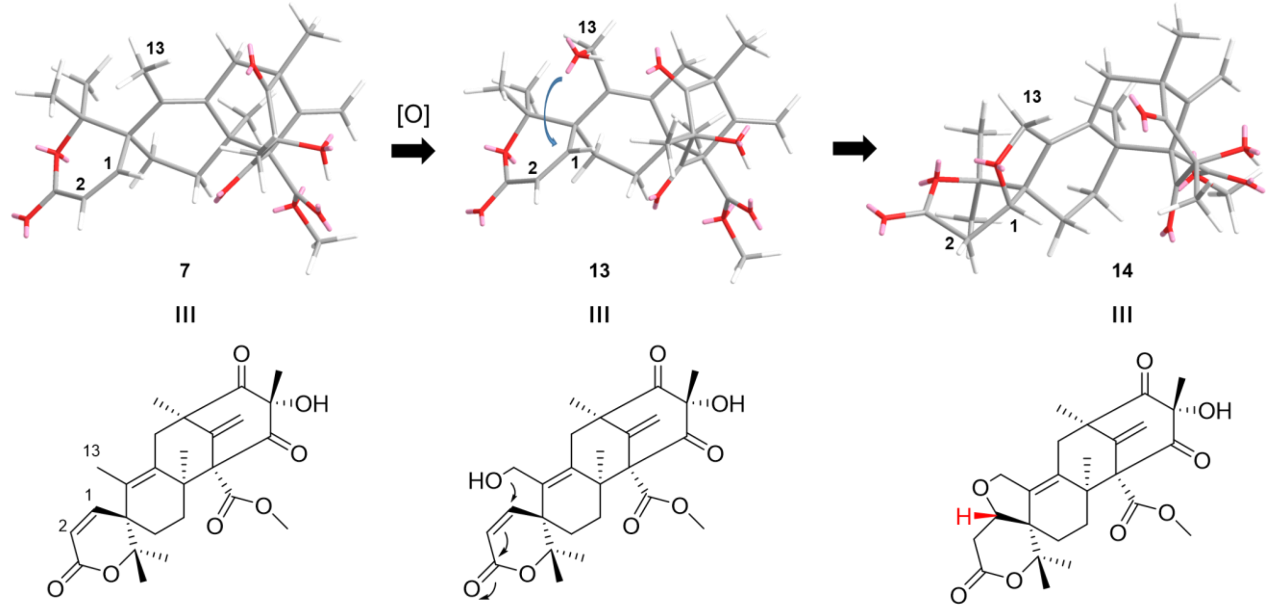

Supplementary Figure 18. Predicted stereochemistry of C-1 based on molecular modeling. The C-1 chiral center in **14** is predicted to have *S* configuration based on the molecular models of **7**, **13** and **14** generated and energy minimized using ChemBio3D Ultra (14.0 MM2).

Supplementary Tables

Supplementary Table 1. Data collection and refinement statistics for the x-ray structure of AusE in the absence of substrate.

	AusE-Mn	AusE- Mn/αKG
**Data collection**		
Wave length (Å)	1.8926	1.1
Space group	$P4_22_12$	$C222$
Cell dimensions		
a, *b*, *c* (Å)	148.1, 148.1, 59.6	220.3, 223.9, 53.7
α, β, γ (°)	90.0, 90.0, 90.0	90.0, 90.0, 90.0
No. reflections	16,664	74,961
Resolution (Å)	49-2.8 (2.9-2.8) *	48-2.1 (2.14-2.1)
R_{merge}	15.8 (119.5)	15.3 (58.1)
$I / \sigma I$	34.4 (6.5)	7.4 (2.4)
Completeness (%)	98.8 (97.5)	98.6 (96.6)
Redundancy	72.7 (77.8)	6.8 (7.2)
**Refinement**		
Resolution (Å)		48-2.1
R_{work} / R_{free}		21.4/26.6
No. atoms		
Protein		7,863
Ligand/ion		24
Water		264
B-factors		
Protein		29.3
Ligand/ion		35.1
Water		30.0
R.m.s. deviations		
Bond lengths (Å)		0.008
Bond angles (°)		0.977
Ramachandran plot (%)※		95.6/4.4/0.0
Accession code		5YBL

*Values in parentheses are for highest-resolution shell.

※Number of residues in favored region / allowed region / outlier region.

Supplementary Table 2. Data collection and refinement statistics for the x-ray structure of PrhA in the absence of substrate.

	PrhA	PrhA-Fe/αKG
**Data collection**		
Wave length (Å)	1.1	1.1
Space group	$P6_2$	$P6_2$
Cell dimensions		
a, b, c (Å)	172.8, 172.8, 44.7	172.4, 172.4, 45.3
α, β, γ (°)	90.0, 90.0, 120.0	90.0, 90.0, 120.0
No. reflections	44,058	44,964
Resolution (Å)	45-2.1 (2.2-2.1) *	43-2.1 (2.2-2.1)
R_{merge}	10.0 (43.7)	4.4 (45.9)
$I / \sigma I$	9.1 (3.2)	17.1 (2.4)
Completeness (%)	99.8 (98.1)	99.6 (98.7)
Redundancy	5.7 (5.6)	3.4 (3.3)
**Refinement**		
Resolution (Å)	43-2.1	43-2.1
R_{work} / R_{free}	18.8/22.9	18.6/22.5
No. atoms		
Protein	4,446	4,482
Ligand/ion	0	22
Water	328	327
B-factors		
Protein	30.4	35.6
Ligand/ion	0	36.8
Water	33.5	38.7
R.m.s. deviations		
Bond lengths (Å)	0.007	0.006
Bond angles (°)	0.828	0.810
Ramachandran plot (%)※	98.1/1.9/0.0	97.6/2.4/0.0
Accession code	5YBM	5YBN

*Values in parentheses are for highest-resolution shell.

※Number of residues in favored region / allowed region / outlier region.

Supplementary Table 3. Data collection and refinement statistics for the x-ray structure of wt and V150L/A232S PrhA solved in the presence of substrates.

	PrhA-Fe/αKG/**5**	PrhA-V150L/A232S- Fe/αKG/**5**	PrhA-V150L/A232S-Fe/αKG/**6**
**Data collection**			
Wave length (Å)	0.98	1.1	0.98
Space group	$P6_2$	$P6_2$	$P6_2$
Cell dimensions			
a, *b*, *c* (Å)	171.8, 171.8, 45.7	171.8, 171.8, 45.9	171.4, 171.4, 45.6
α, β, γ (°)	90.0, 90.0, 120.0	90.0, 90.0, 120.0	90.0, 90.0, 120.0
No. reflections	39,266	33,590	36,762
Resolution (Å)	44-2.2 (2.3-2.2) *	50-2.3 (2.4-2.3)	49-2.25 (2.32-2.25)
R_{merge}	4.4 (57.3)	5.0 (44.8)	6.5 (61.4)
$I / \sigma I$	17.4 (2.1)	15.2 (2.6)	14.3 (2.6)
Completeness (%)	99.6 (98.8)	97.9 (96.4)	99.8 (99.4)
Redundancy	3.4 (3.4)	3.2 (3.2)	5.0 (5.0)
**Refinement**			
Resolution (Å)	44-2.2	50-2.3	44-2.25
R_{work} / R_{free}	21.3/26.2	21.9/26.4	20.7/25.5
No. atoms			
Protein	4,276	4,235	4,346
Ligand/ion	55	55	88
Water	183	160	191
B-factors			
Protein	46.1	43.2	40.5
Ligand/ion	58.0	55.2	43.7
Water	44.7	41.3	39.6
R.m.s. deviations			
Bond lengths (Å)	0.008	0.007	0.008
Bond angles (°)	0.938	0.945	0.942
Ramachandran plot (%)※	96.5/3.6/0.0	97.5/2.5/0.0	96.7/3.3/0.0
Accession code	5YBO	5YBP	5YBQ

*Values in parentheses are for highest-resolution shell.

※Number of residues in favored region / allowed region / outlier region.

Supplementary Table 4. Data collection and refinement statistics for the x-ray structure of V150L/A232S and V150L/A232S/M241V PrhA solved in the presence of substrates.

	PrhA-V150L/A232S- Fe/αKG/**7**	PrhA-V150L/A232S- Fe/αKG/**3**	PrhA-V150L/A232S/M241V- Fe/αKG/**3**
**Data collection**			
Wave length (Å)	1.1	1.1	1.1
Space group	$P6_2$	$P6_2$	$P6_2$
Cell dimensions			
a, b, c (Å)	171.4, 171.4, 45.7	171.5, 171.5, 45.8	171.6, 171.6, 45.6
α, β, γ (°)	90.0, 90.0, 120.0	90.0, 90.0, 120.0	90.0, 90.0, 120.0
No. reflections	36,185	34,292	32,026
Resolution (Å)	44-2.26 (2.33-2.26) *	49-2.3 (2.4-2.3)	50-2.35 (2.43-2.35)
R_{merge}	4.7 (52.1)	5.0 (48.9)	5.8 (54.5)
$I / \sigma I$	14.9 (2.2)	14.7 (2.4)	13.8 (2.0)
Completeness (%)	99.4 (98.3)	98.9 (99.3)	98.6 (97.1)
Redundancy	3.2 (3.2)	3.5 (3.6)	3.5 (3.7)
**Refinement**			
Resolution (Å)	44-2.26	49-2.3	50-2.35
R_{work} / R_{free}	21.1/25.9	21.3/26.9	21.7/26.4
No. atoms			
Protein	4,310	4,304	4,341
Ligand/ion	88	54	86
Water	176	119	162
B-factors			
Protein	45.2	45.8	45.4
Ligand/ion	57.0	55.7	47.8
Water	44.2	42.0	45.5
R.m.s. deviations			
Bond lengths (Å)	0.008	0.008	0.007
Bond angles (°)	1.058	0.923	0.931
Ramachandran plot (%)※	96.8/3.2/0.0	95.4/4.6/0.0	96.7/3.3/0.0
Accession code	5YBR	5YBS	5YBT

*Values in parentheses are for highest-resolution shell.

※Number of residues in favored region / allowed region / outlier region.

Supplementary Table 5. Distance table.

Distance	Chain A	Chain B	Average
**PrhA Wild-type Fe/AKG/5 (Fig. 5b) (PDB ID: 5YBO)**			
Fe – C2(5)	-	4.4	4.4
Fe – C5(5)	-	5.2	5.2
CG2 V150 – O3(5)	-	3.5	3.5
CB A232 – O3(5)	-	3.6	3.6
SD M241 – C13(5)	-	4.8	4.8
**PrhA V150L/A232S Fe/AKG/5 (Fig. 5c) (PDB ID: 5YBP)**			
Fe – C2(5)	-	4.2	4.2
Fe – C5(5)	-	5.2	5.2
CD1 L150 – O3(5)	-	3.4	3.4
CD2 L150 – O3(5)	-	3.1	3.1
OG S232 – O3(5)	-	2.9	2.9
SD M241 – C13(5)	-	4.7	4.7
**PrhA V150L/A232S Fe/AKG/6 (Fig. 5d) (PDB ID: 5YBQ)**			
Fe – C5(6)	5.3	5.2	5.3
Fe – C9(6)	5.9	6.0	6.0
CD1 L150 – O3(6)	4.0	3.3	3.7
CD2 L150 – O3(6)	3.8	3.3	3.6
OG S232 – O3(6)	2.8	2.6	2.7
SD M241 – C13(6)	4.1	3.6	3.9
**PrhA V150L/A232S Fe/AKG/7 (Fig. 5e) (PDB ID: 5YBR)**			
Fe – C13(7)	7.4	7.4	7.4
CD1 L150 – O3(7)	3.9	3.6	3.8
CD2 L150 – O3(7)	3.8	3.5	3.7
OG S232 – O3(7)	2.8	2.8	2.8
SD M241 – C13(7)	3.4	3.7	3.6
**PrhA V150L/A232S/M241V Fe/AKG/3 (Fig. 5f) (PDB ID: 5YBT)**			
Fe – C3(3)	4.8	4.7	4.8
Fe – C5(3)	5.4	5.2	5.3
CD1 L150 – O3(3)	4.5	4.7	4.6
CD2 L150 – O3(3)	4.3	4.5	4.4
OG S232 – O3(3)	2.7	2.4	2.6

CG1 V241 – C13(3)	(nd)#	4.5	4.5
CG2 V241 – C13(3)	(nd)	4.2	4.2
**PrhA V150L/A232S Fe/AKG/3 (Fig. S14) (PDB ID: 5YBS)**			
Fe – C3(3)	-	4.7	4.7
Fe – C5(3)	-	5.3	5.3
CD1 L150 – O3(3)	-	4.9	4.9
CD2 L150 – O3(3)	-	4.1	4.1
OG S232 – O3(3)	-	2.4	2.4
SD M241 – C13(3)	-	3.7	3.7

nd: no density was observed for the side chain.

Supplementary Table 6. Primers used in this study.

Primer	Sequence
ausE-6-298-f	5′-GGAGATATACATATGCCATCCCGTCTCCAGAAATTTC-3′
ausE-6-298-r	5′-CGCGGATCCCTAAGCGCTGGAGATCAAGCC-3′
prhA-6-298-f	5′-GGAGATATACATAGCTCCAGAGATTCCCCGCCAC-3′
prhA-6-298-r	5′-CGCGGATCCCTAAGCGCTGGAGATCAACCCAAGATGATGAGCAATATCC-3′
prhA-V150L-f	5’-GAGTGTCTCATCAACTTTTTCTGCG-3’
prhA-V150L-r	5’-GTTGATGAGACACTCAGGCCCG-3’
prhA-A232S-f	5’-TGCGAATAATCGACAGGGCAAGCG-3’
prhA-A232S-r	5’-CGCTTGCCCTGTCGATTATTCGCA-3’
prhA-M241V-f	5’-ACTGCGTCCCGTGCAGGCTTTCT-3’
prhA-M241V-r	5’-AGAAAGCCTGCACGGGACGCAGT-3’
ausE-L150V-f	5’-GAGTGCGTCGTCAACTTCTTCTGCG-3’
ausE-L150V-r	5’-GTTGACGACGCACTCGGGAGCATC-3’
ausE-S232A-f	5’-GCGTTTGCCACGATCCGCCGAGAAC-3’
ausE-S232A-r	5’-GATCGTGGCAAACGCCAGCGCG-3’)

Supplementary Note 1.

HRMS analysis of compound 13

HRMS analysis revealed the molecular formula of compound **13** to be $C_{26}H_{32}O_8$, indicating the presence of additional oxygen atom compared to **7**. HRMS (m/z): [M+H]+ calcd. for $C_{26}H_{33}O_8$, 473.5354; found, 473.5415.

Structural elucidation of compound 14

HRMS analysis revealed the molecular formula of compound **14** to be $C_{26}H_{32}O_8$ and its NMR data were similar to those of **7**, including spiro-lactone ring system. However, signals corresponding to allylic methyl group (C-13) and conjugated olefin moiety A-ring (C-1 and C-2) observed in 8 were absent in the NMR spectra of **14**. Instead, NMR data indicated the presence of one oxymethylene (C-11, δ_H 4.24, δ_C 68.9 ppm), oxymethine (C-1, δ_H 3.65 ppm, δ_C 84.5 ppm) and aliphatic methylene group (C-2, δ_H 2.87 and 2.71 ppm, δ_C 33.8 ppm), suggesting that allylic methyl group of **7** was oxidated. The planer structure was constructed based on the 2D NMR spectra including COSY, HSQC and HMBC. The presence of ether ring (E-ring) in **14** was confirmed based on the HMBC correlations of H-13/C-1 and downfield-shifted δ_C value at C-1 position (84.5 ppm).

Considering **14** was directly converted from **7**, stereochemistries of **14** should be identical to those of **7** except for the newly generated chiral center at C-1 position. Although NOESY analysis did not conclude the stereochemistry at C-1 position, molecular modeling (Chem3D Ultra 14.0, MM2) of **7** and putative precursor **13** predicted whether the primary alcohol in **13** to attack from either side of C-1 position (Supplementary Figure 18), and the configuration of C-1 position expect to be *S* based on these observations.

Compound **14**: ^{1}H NMR (900 MHz, $CDCl_3$): δ 5.54 (d, 1H, J = 1.0 Hz, H-1’), 5.04 (d, 1H, J = 1.0 Hz, H-1’), 4.24 (s, 2H, H-13), 3.80 (s, 3H, H-1”), 3.65 (t, 1H, J = 3.5 Hz, H-1), 2.87 (dd, 1H, J = 3.5, 17.5 Hz, H-2), 2.84 (ddd, 1H, J = 2.5, 4.5, 14.0 Hz, H-7), 2.71 (dd, 1H, J = 3.5, 17.5 Hz, H-2), 2.63 (d, 1H, J = 14.0 Hz, H-11), 2.27 (d, 1H, J = 14.0 Hz, H-11), 1.94 (ddd, 1H, J = 2.5, 4.5, 14.5 Hz, H-6), 1.73 (ddd, 1H, J = 4.5, 14.0, 14.0

Hz, H-7), 1.56 (s, 3H, H-12), 1.54 (s, 3H, H-9'), 1.45 (s, 3H, H-14), 1.31 (s, 3H, H-10'), 1.30 (s, 3H, H-15), 0.99 (ddd, 1H, J = 4.5, 14.0, 14.0 Hz, H-6). ^{13}C NMR (225 MHz, $CDCl_3$): δ 206.2 (C-4'), 203.5 (C-6'), 170.1 (C-3), 169.3 (C-8'), 145.0 (C-2'), 138.9 (C-10), 130.6 (C-9), 114.2 (C-1'), 84.5 (C-1), 84.2 (C-4), 78.6 (C-5'), 72.2 (C-7'), 68.9 (C-13), 52.6 (C-1"), 51.4 (C-3'), 46.6 (C-5), 43.4 (C-8), 41.9 (C-11), 33.8 (C-2), 30.5 (C-7), 29.4 (C-15), 27.6 (C-14), 27.0 (C-6), 26.3 (C-12), 21.5 (C-9'), 15.5 (C-10'). HRMS (*m/z*): $[M+H]^+$ calcd. for $C_{26}H_{33}O_8$, 473.5354 ; found, 473.5415.

Structural elucidation of compound 15

HRMS analysis revealed the molecular formula of compound **15** to be $C_{26}H_{32}O_9$, indicating the presence of additional oxygen atom compared to **14**. ^{1}H and ^{13}C NMR were nearly identical to those of **14**, but 1D NMR spectra lacked the signal for C-1 oxymethine moiety observed in **14**. On the other hand, ^{13}C NMR spectrum showed new quaternary carbon signal at 104.1 ppm, suggesting that C-1 position of **14** was oxygenated and converted to hemiacetal group. It was supported by the HMBC correlations of H-2/C-1 and H-13/C-1, the down-field shifts of chemical shifts of C-2 (**15**: 40.7 ppm, **14**: 33.8 ppm) and C-5 (**15**: 51.5 ppm, **14**: 46.6 ppm) position. Because **15** was directly converted from **14** by enzymatic reaction, these compounds should share the same stereochemistries and the configuration of C-1 position was thought to be *S*.

Compound **15** ^{1}H NMR (900 MHz, $CDCl_3$):δ 5.53 (s,1H, H-1'), 5.03 (s, 1H, H-1'), 4.37 (d, 1H, J = 13.0 Hz, H-13), 4.25 (dd, 1H, J = 2.5, 13.0 Hz H-13), 3.80 (s, 3H, H-1"), 2.89 (d, 1H, J = 17.0 Hz, H-2), 2.84 (m, 1H, H-7), 2.82 (d, 1H, J = 17.0Hz, H-2), 2.64 (d, 1H, J = 13.0, H-11), 2.22 (d, 1H, J = 13.0, H-11), 1.67 (m, 1H, H-7), 1.66 (m, 1H, H-6), 1.61 (m, 1H, H-6), 1.55 (s, 3H, H-12), 1.53 (s, 3H, H-9'), 1.50 (s, 3H, H-14), 1.35 (s, 3H, H-10'), 1.30 (s, 3H, H-15), 0.99 (ddd, 1H, J = 4.5, 14.0, 14.0, H-6). ^{13}C NMR (225 MHz, $CDCl_3$): δ 206.6 (C-4'), 202.6 (C-6'), 169.5 (C-3), 169.4 (C-8'), 145.6 (C-2'), 137.2 (C-10), 131.2 (C-9), 113.7 (C-1'), 104.1 (C-1), 84.3 (C-4), 79.2 (C-5'), 72.1 (C-7'), 68.8 (C-13), 52.6 (C-1"), 51.5 (C-5), 51.4 (C-3'), 43.4 (C-8), 42.2 (C-11), 40.7 (C-2), 29.9 (C-7), 29.9 (C-15), 27.8 (C-14), 26.2 (C-12), 22.1 (C-6), 21.7 (C-9'), 14.6 (C-10'). HRMS (*m/z*): $[M+H]^+$ calcd. for $C_{26}H_{33}O_9$, 489.5348 ; found, 489.5405.

Structural elucidation of compound 16

HRMS analysis revealed the molecular formula of compound **16** to be $C_{26}H_{30}O_9$. ^1H NMR spectrum showed very similar patterns to those of **7**, including the signals corresponding to C-1/C-2 double bond (δ_H 6.57 ppm (H-1) and 5.93 ppm (H-2)). On the other hand, ^1H NMR data indicated that vinyl methyl group of C-13 position was absent in **16** as in the case of **15** and **14**. Because of low quantity of **16** and several signal broadening in NMR data, we could not assigned carbon resonances corresponding to C-1, C-10 and C-13, although the large parts of planer structure was fully elucidated by analyzing 1D (^1H and ^{13}C) and 2D (COSY, HSQC and HMBC) NMR data set. However, these spectroscopic data and the fact **16** was enzymatically converted from **13** or **7** (not from **15** and **14**) strongly indicated that **16** is the analogue of **7** whose C-13 methyl group was oxidized to carboxylic acid. It was further confirmed that **16** was converted to corresponding methyl ester by treating with TMS-diazomethane.

Compound **16** ^1H NMR (900 MHz, CD_3OD):δ 6.57 (brs, 1H, H-1), 5,93 (d, 1H, J = 9.5), 5.45 (s,1H, H-1'), 4.94 (s, 1H, H-1'), 3.71 (s, 3H, H-1''), 2.75 (brd, 1H, J = 15.0 Hz, H-11), 2.67 (m, 1H, H-7), 2.53 (brd, 1H, J = 15.0Hz, H-11), 2.05 (m, 1H, H-7), 1.79 (m, 1H, H-6), 1.62 (s, 3H, H-14), 1.61 (m, 1H, H-6), 1.52 (s, 3H, H-12), 1.43 (s, 3H, H-9'), 1.40 (s, 3H, H-15), 1.38 (s, 3H, H-10'). ^{13}C NMR (225 MHz, CD_3OD): δ 207.1 (C-4'), 205.2 (C-6'), 169.5 (C-8'), 143.1 (C-2'), 132.7* (C-9), 118.9* (C-2), 112.8 (C-1'), 86.1 (C-4), 76.4 (C-5'), 71.6 (C-7'), 51.3 (C-3'), 50.9 (C-1''), 45.2* (C-5), 44.8 (C-8), 42.5 (C-11), 25.5* (C-6), 24.5 (C-7), 23.9 (C-15), 22.4 (C-14), 22.0 (C-12), 20.4 (C-9'), 17.6 (C-10') (*The chemical shifts were determined based on HMBC and HSQC correlations. ** carbon resonances corresponding to C-1, C-10 and C-13 were not assigned due to low quantity of **21** and signal broadening.). HRMS (*m/z*): $[M+H]^+$ calcd. for $C_{26}H_{31}O_9$, 487.5189 ; found, 487.5245.

Methyl esterification of 16

A small part of **16** (ca. 30 μg) was dissolved in 200 μl of methanol and excess amount of TMS-diazomethane (10 μl of 0.6 M hexane solution) was added and the mixture was stirred for 1 h at room

temperacture. The reaction was quenched by removing TMS-diazomethane by evaporation and the resulting residue was re-dissolved in 50 µl of methanol for LC-HRMS analysis.

a

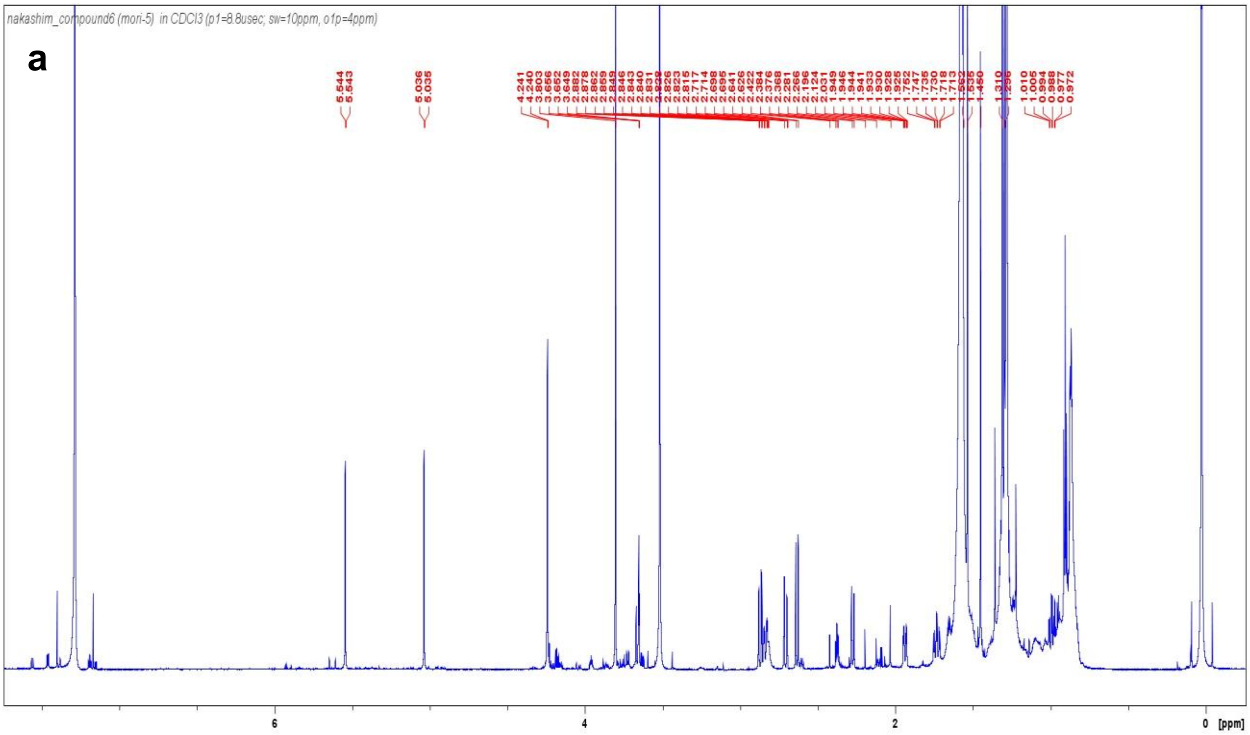
nakashim_compound6 (mori-5) in CDCl3 (p1=8.8usec; sw=10ppm, o1p=4ppm)
[ppm]

b

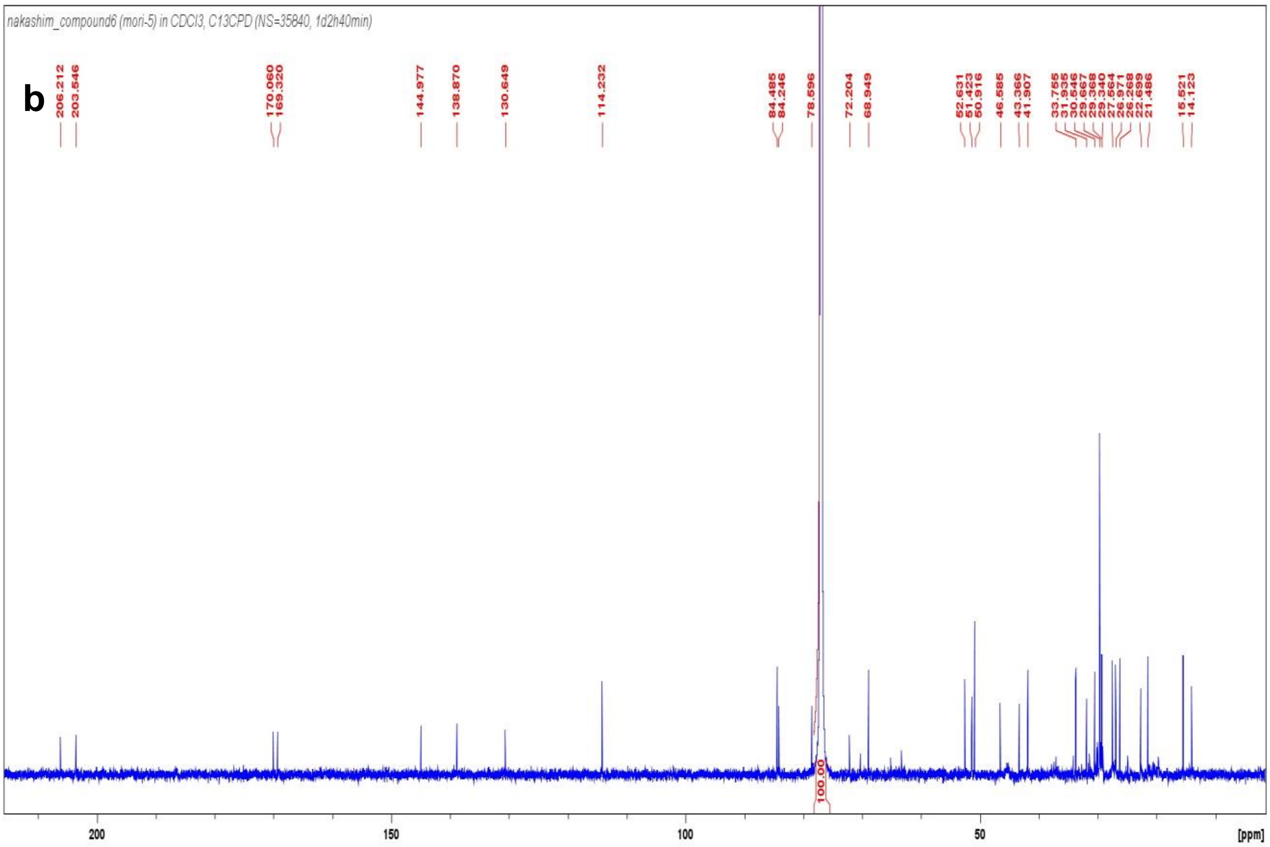
nakashim_compound6 (mori-5) in CDCl3, C13CPD (NS=35840, 1d2h40min)
[ppm]

c

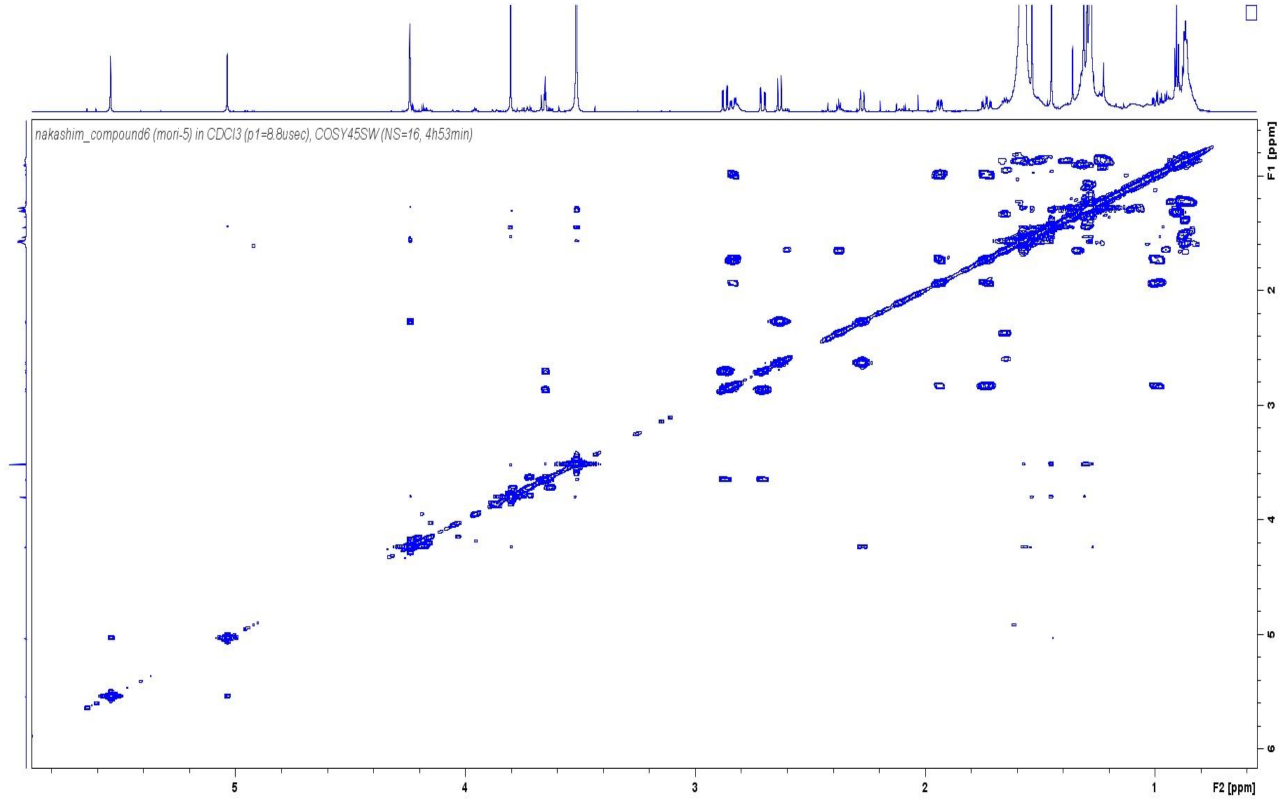

nakashim_compound6 (mori-5) in CDCl3 (p1=8.8usec), COSY45SW (NS=16, 4h53min)
F1 [ppm]
F2 [ppm]

d

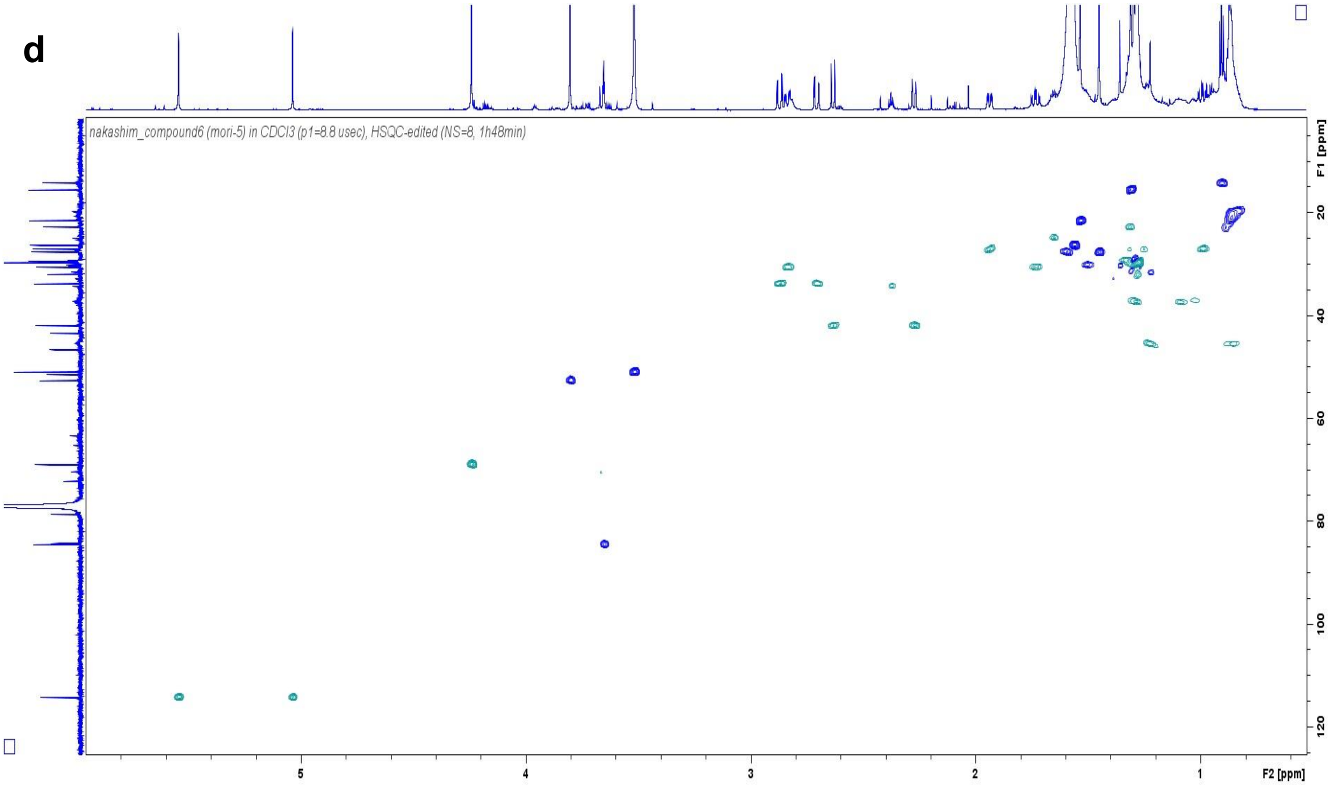

nakashim_compound6 (mori-5) in CDCl3 (p1=8.8 usec), HSQC-edited (NS=8, 1h48min)
F1 [ppm]
F2 [ppm]

e

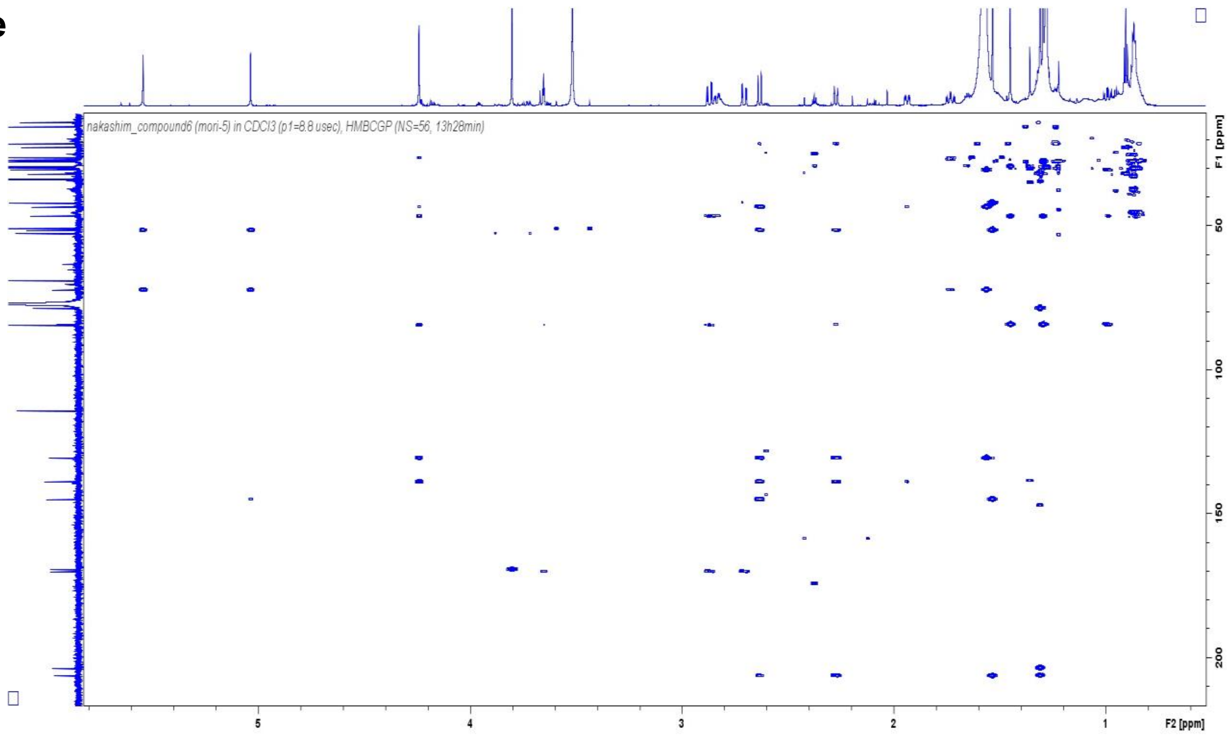

f

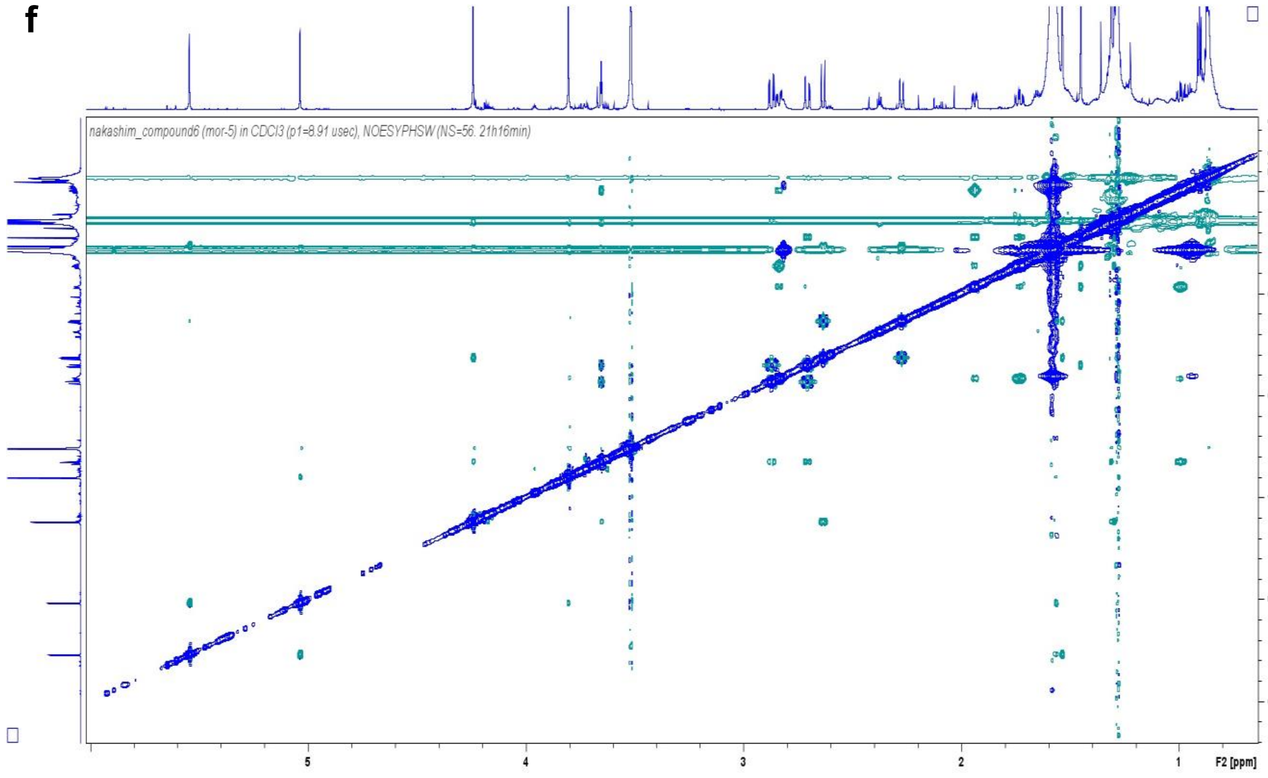

NMR spectra of compound **14**. (a) ^{1}H NMR (900 MHz), (b) ^{13}C NMR (225 MHz), (c) COSY (900 MHz), (d) HSQC (900 MHz), (e) HMBC (900 MHz), (f) NOESY (900 MHz). All data were measured in $CDCl_3$.

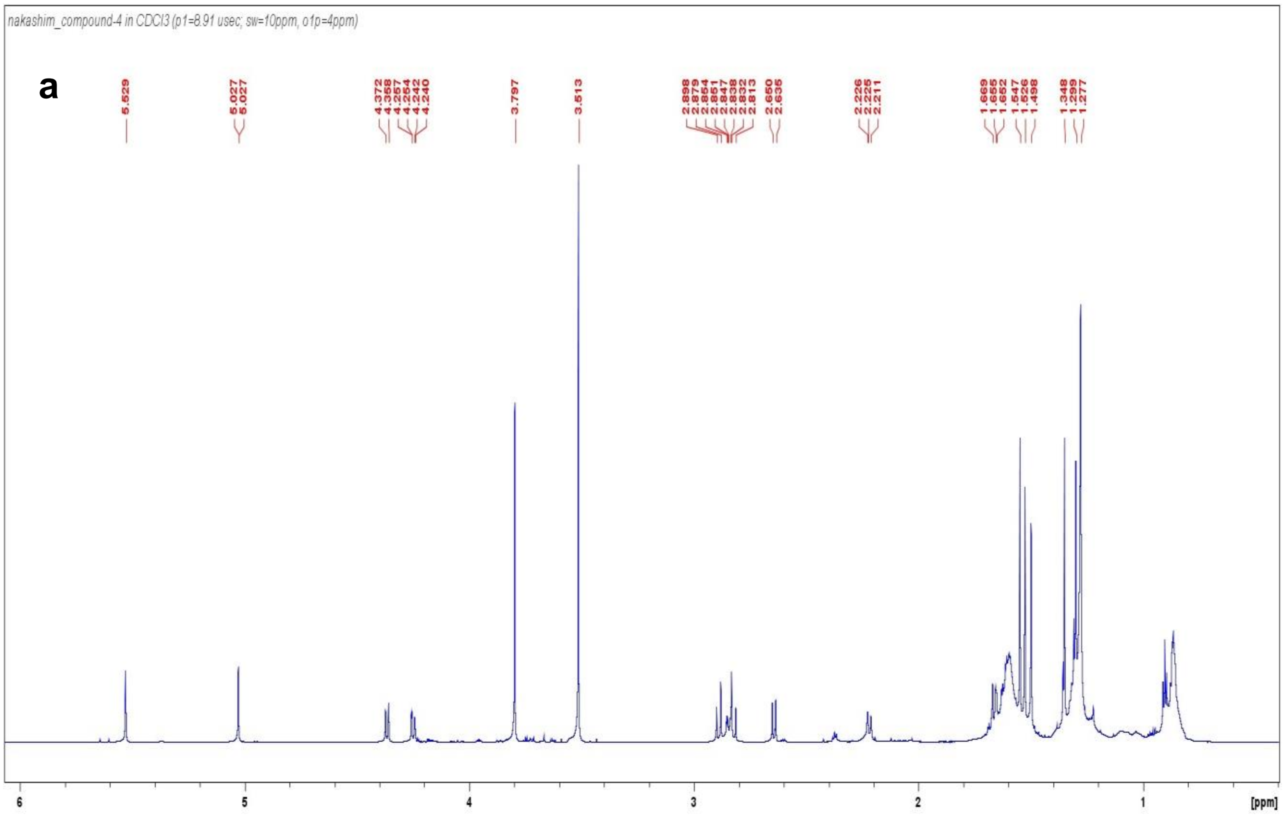
nakashim_compound-4 in CDCl3 (p1=8.91 usec; sw=10ppm, o1p=4ppm)
a
5.529
5.027
5.027
4.372
4.358
4.257
4.251
4.242
4.240
3.797
3.513
2.898
2.879
2.854
2.851
2.847
2.838
2.832
2.813
2.650
2.635
2.226
2.225
2.211
1.669
1.655
1.652
1.547
1.526
1.498
1.348
1.299
1.277
[ppm]

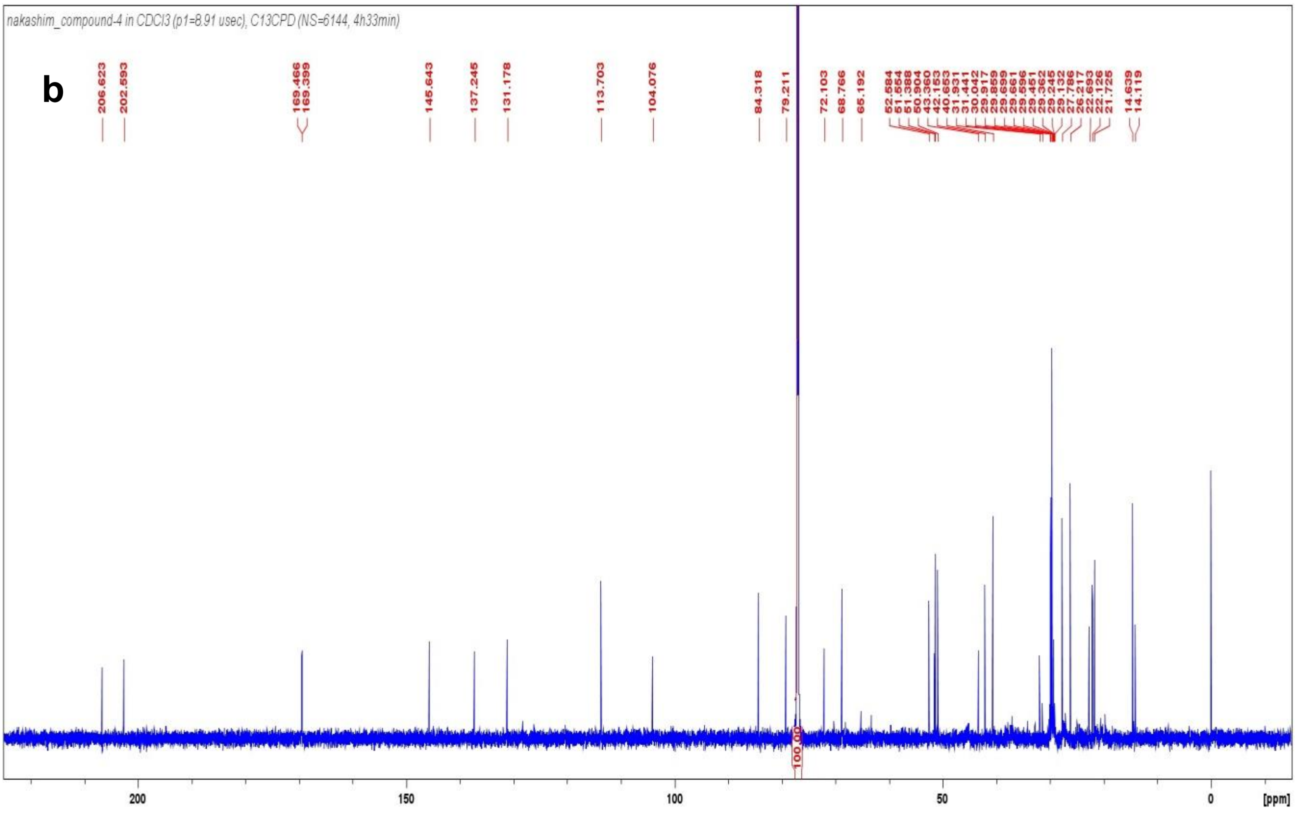
nakashim_compound-4 in CDCl3 (p1=8.91 usec), C13CPD (NS=6144, 4h33min)
b
206.623
202.593
169.466
169.399
145.643
137.245
131.178
113.703
104.076
84.318
79.211
72.103
68.766
65.192
[ppm]

c

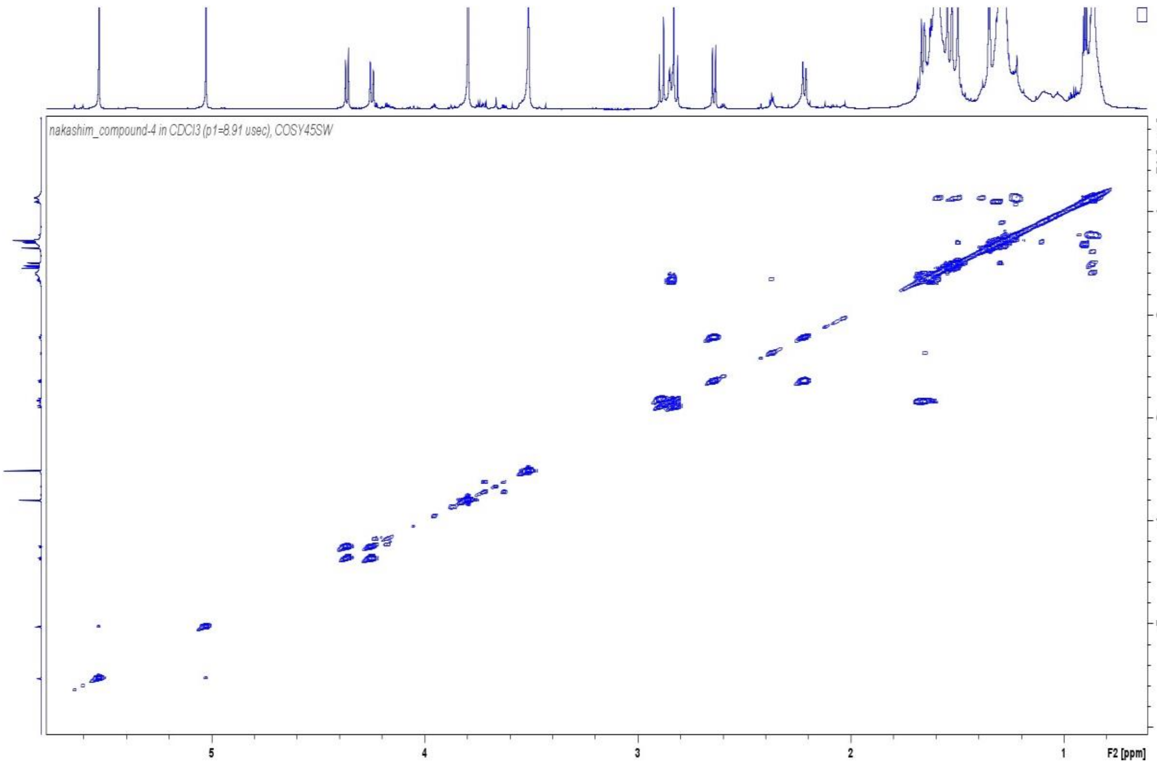
nakashim_compound-4 in CDCl3 (p1=8.91 usec), COSY45SW
F1 [ppm]
F2 [ppm]
1
2
3
4
5

d

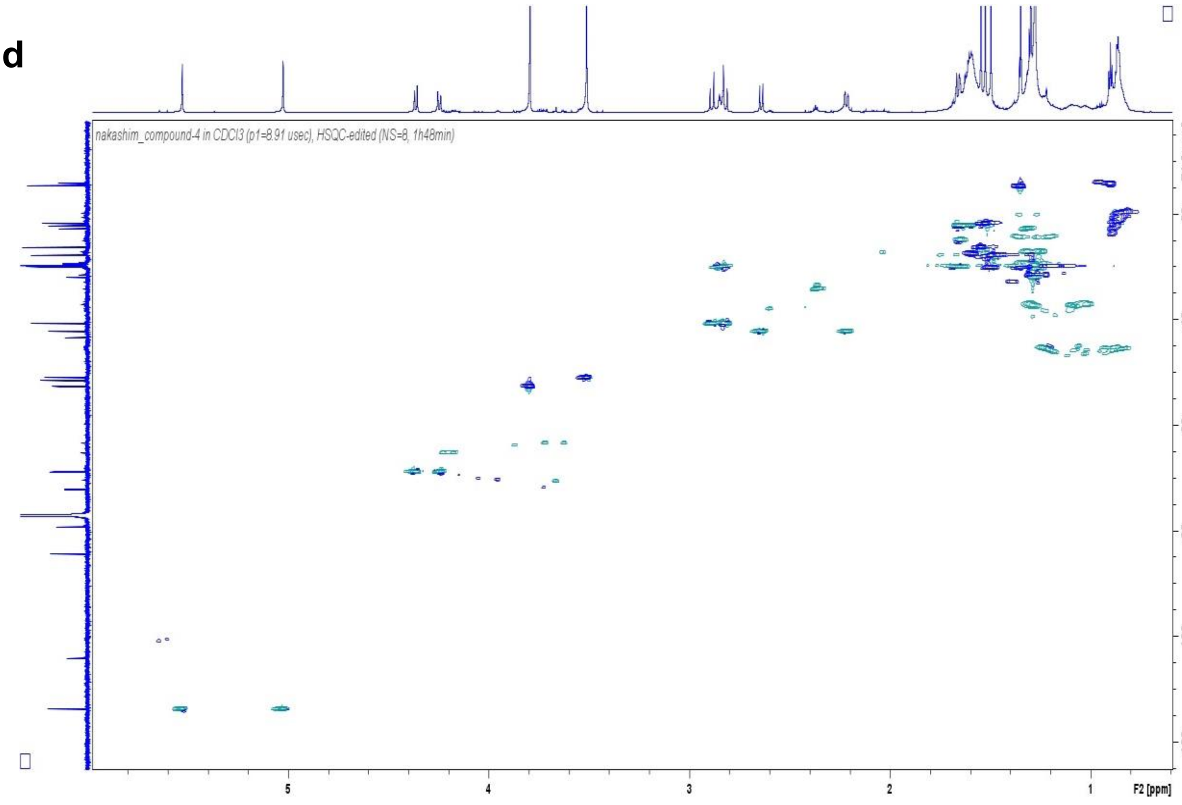
nakashim_compound-4 in CDCl3 (p1=8.91 usec), HSQC-edited (NS=8, 1h48min)
F1 [ppm]
F2 [ppm]
20
40
60
80
100
120
1
2
3
4
5

e

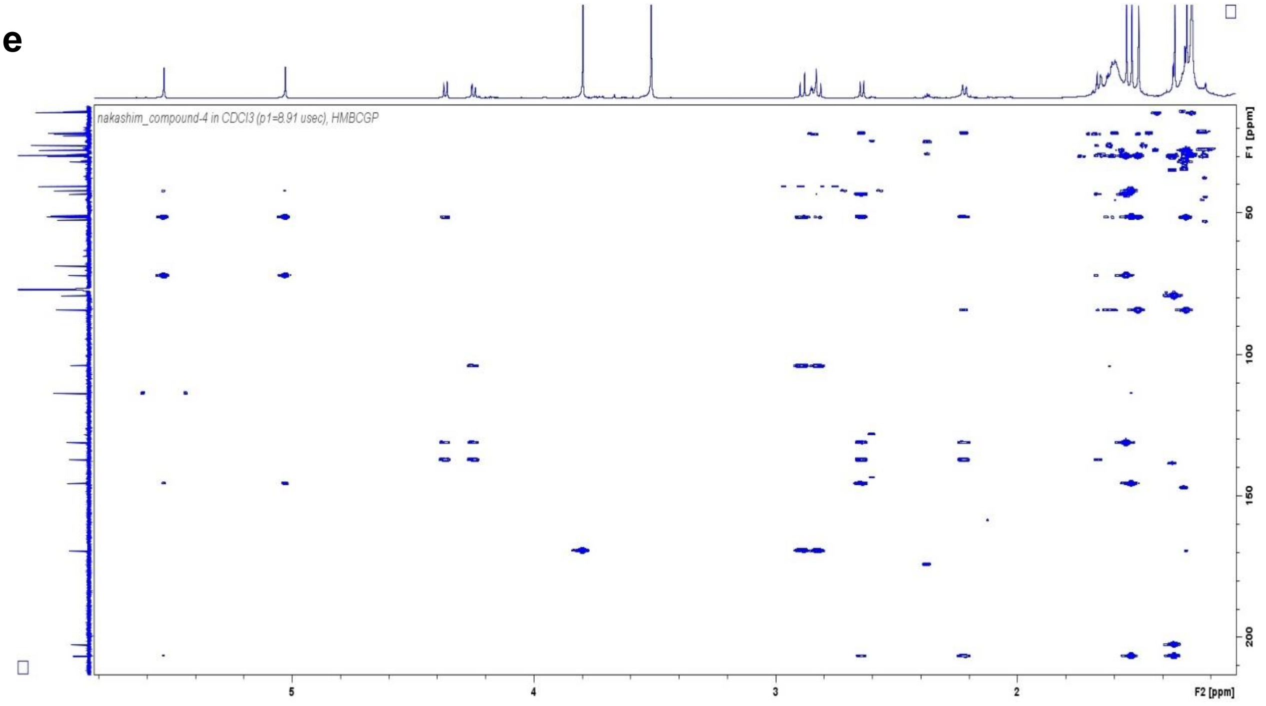

f

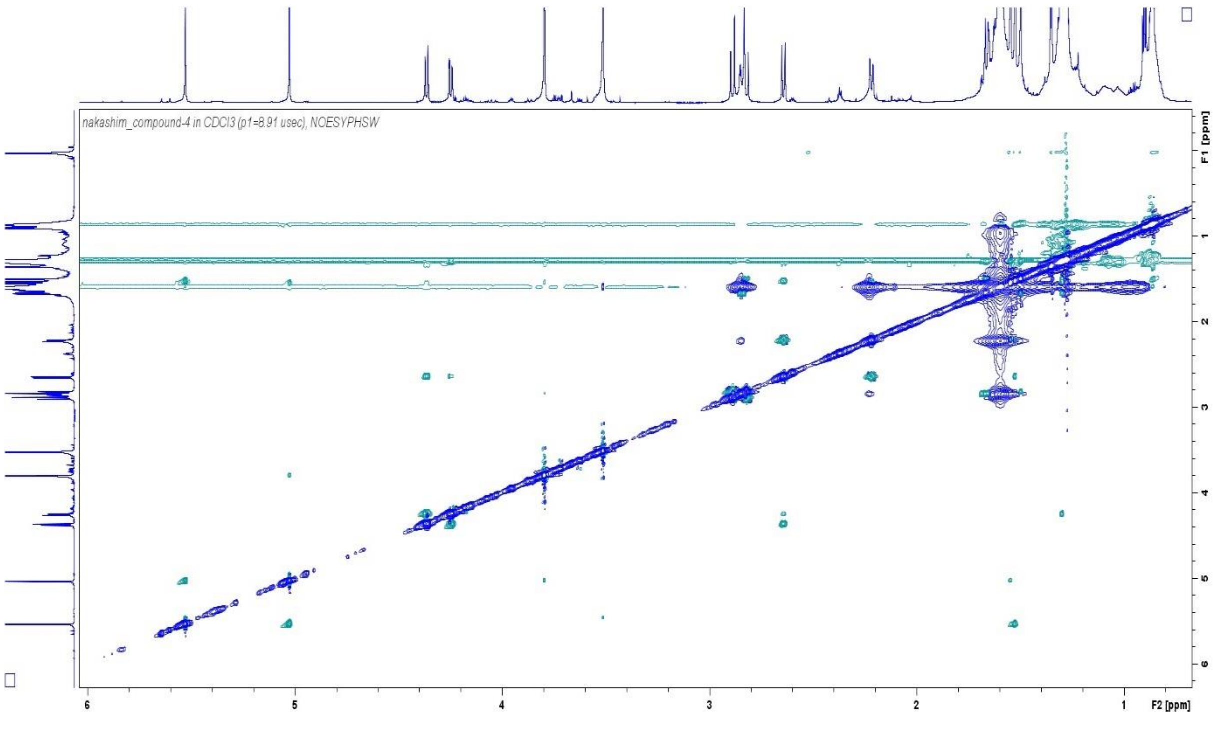

NMR spectra of compound **15**. (a) ^{1}H NMR (900 MHz), (b) ^{13}C NMR (225 MHz), (c) COSY (900 MHz), (d) HSQC (900 MHz), (e) HMBC (900 MHz), (f) NOESY (900 MHz). All data were measured in $CDCl_3$.

a

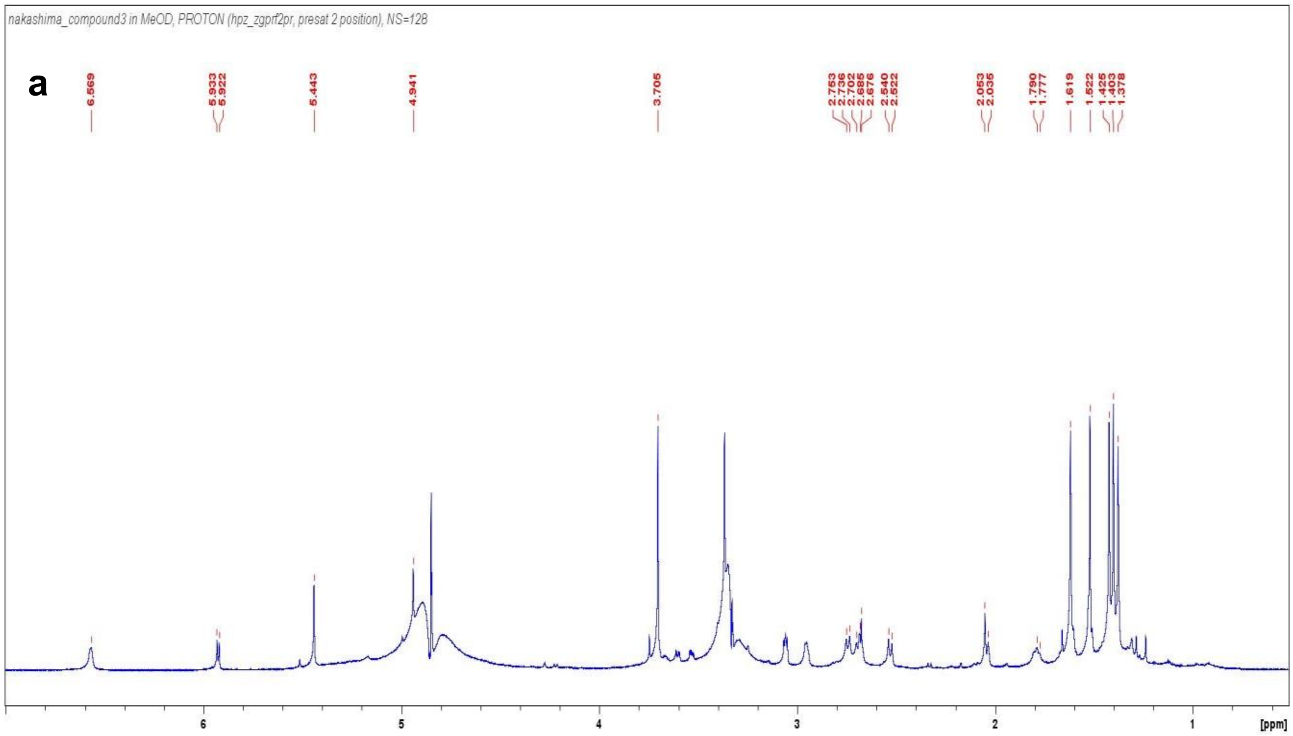
nakashima_compound3 in MeOD, PROTON (hpz_zgprf2pr, presat 2 position), NS=128
6.569
5.933
5.922
5.443
4.941
3.705
2.753
2.736
2.702
2.685
2.676
2.540
2.522
2.053
2.035
1.790
1.777
1.619
1.522
1.425
1.403
1.378
6
5
4
3
2
1
[ppm]

b

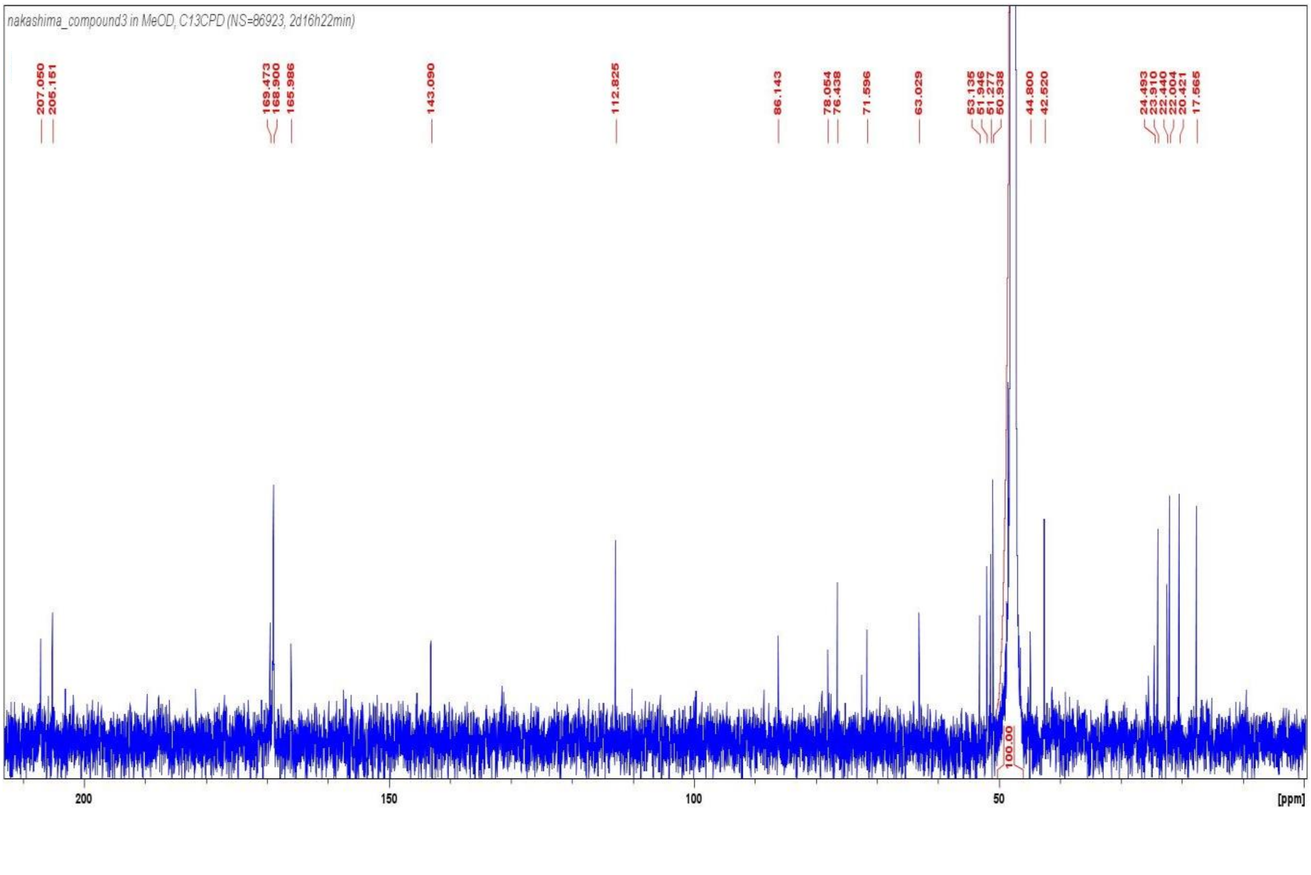
nakashima_compound3 in MeOD, C13CPD (NS=86923, 2d16h22min)
207.050
205.151
169.473
168.900
165.986
143.090
112.825
86.143
78.054
76.438
71.596
63.029
53.135
51.946
51.277
50.938
44.800
42.520
24.493
23.910
22.440
22.004
20.421
17.565
100.00
200
150
100
50
[ppm]

c

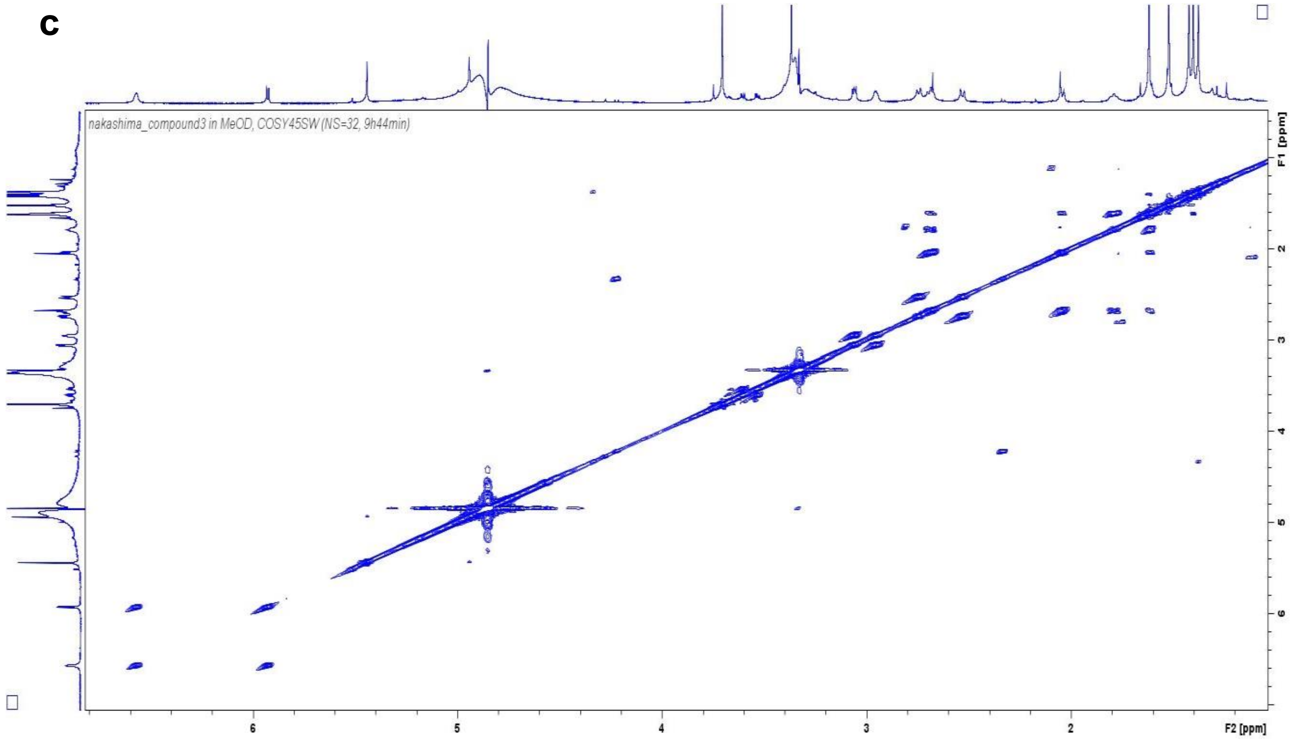
nakashima_compound3 in MeOD, COSY45SW (NS=32, 9h44min)
F1 [ppm]
F2 [ppm]

d

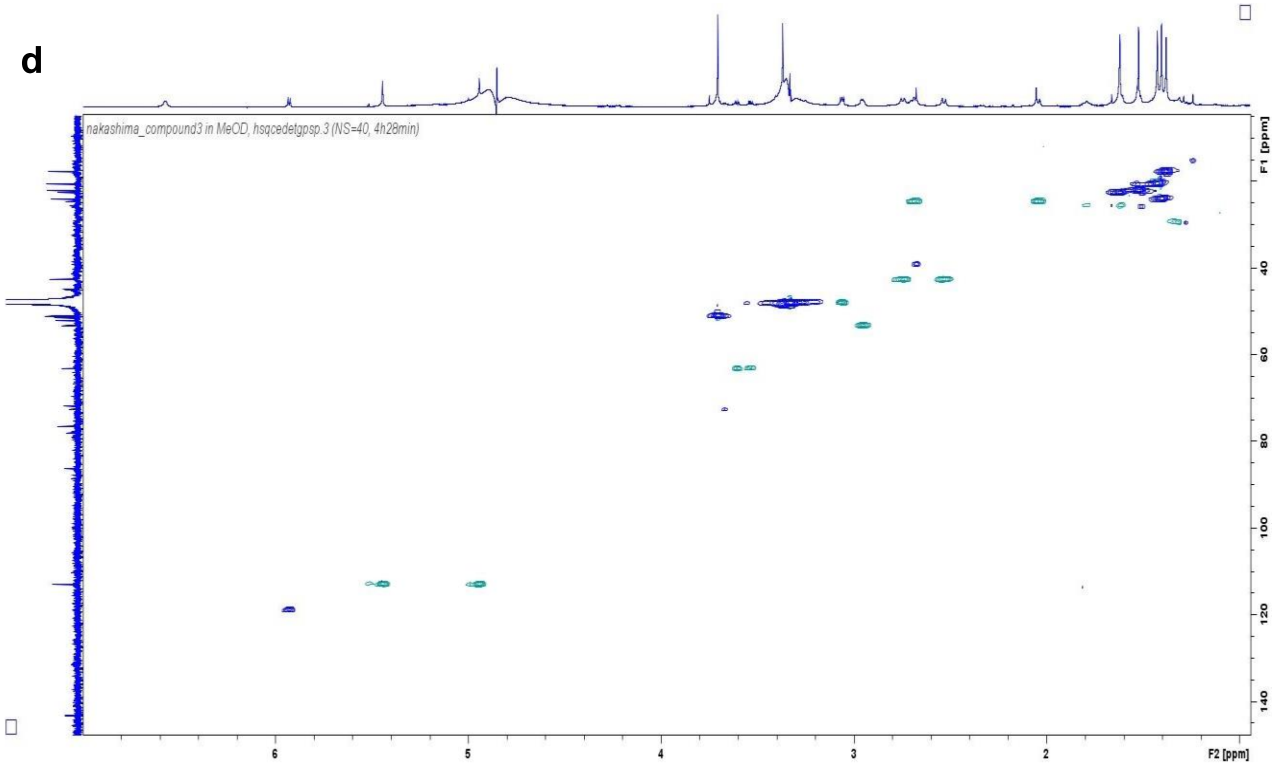
nakashima_compound3 in MeOD, hsqcedetgpsp.3 (NS=40, 4h28min)
F1 [ppm]
F2 [ppm]

e

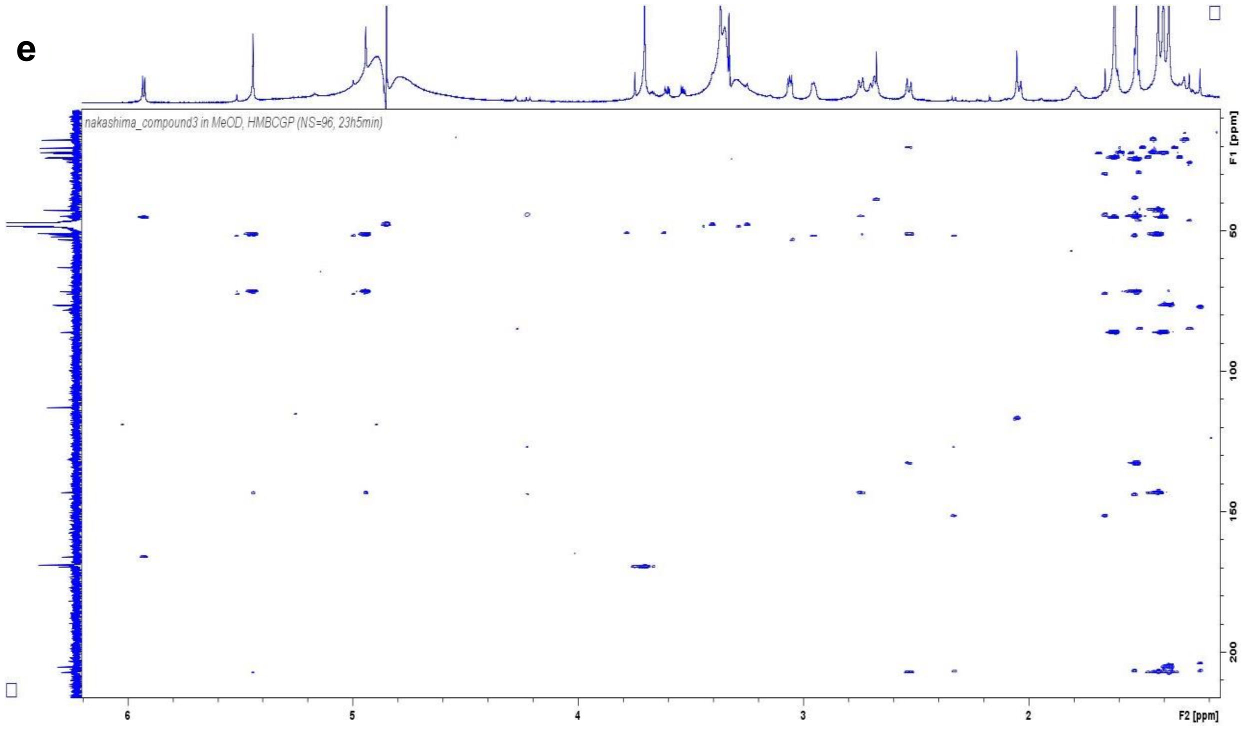

NMR spectra of compound **16**. (a) ^{1}H NMR (900 MHz), (b) ^{13}C NMR (225 MHz), (c) COSY (900 MHz), (d) HSQC (900 MHz), (e) HMBC (900 MHz). All data were measured in CD_3OD.
